# Stochasticity and Determinism: How Density-Independent and Density-Dependent Processes Affect Population Variability

**DOI:** 10.1371/journal.pone.0098940

**Published:** 2014-06-03

**Authors:** Jan Ohlberger, Lauren A. Rogers, Nils Chr. Stenseth

**Affiliations:** Centre for Ecological and Evolutionary Synthesis (CEES), Department of Biosciences, University of Oslo, Oslo, Norway; Texas Tech University, United States of America

## Abstract

A persistent debate in population ecology concerns the relative importance of environmental stochasticity and density dependence in determining variability in adult year-class strength, which contributes to future reproduction as well as potential yield in exploited populations. Apart from the strength of the processes, the timing of density regulation may affect how stochastic variation, for instance through climate, translates into changes in adult abundance. In this study, we develop a life-cycle model for the population dynamics of a large marine fish population, Northeast Arctic cod, to disentangle the effects of density-independent and density-dependent processes on early life-stages, and to quantify the strength of compensatory density dependence in the population. The model incorporates information from scientific surveys and commercial harvest, and dynamically links multiple effects of intrinsic and extrinsic factors on all life-stages, from eggs to spawners. Using a state-space approach we account for observation error and stochasticity in the population dynamics. Our findings highlight the importance of density-dependent survival in juveniles, indicating that this period of the life cycle largely determines the compensatory capacity of the population. Density regulation at the juvenile life-stage dampens the impact of stochastic processes operating earlier in life such as environmental impacts on the production of eggs and climate-dependent survival of larvae. The timing of stochastic versus regulatory processes thus plays a crucial role in determining variability in adult abundance. Quantifying the contribution of environmental stochasticity and compensatory mechanisms in determining population abundance is essential for assessing population responses to climate change and exploitation by humans.

## Introduction

The relative importance of density-independent and density-dependent processes has long been a matter of debate in population ecology [1], [2]. Both stochastic density-independent factors, for instance related to climate variation, and density-dependent processes (e.g. competition for food or habitat) influence the survival of individuals in a population and ultimately determine the variation in population abundance through time. The number of individuals at any life-history stage depends on the survival experienced by all life-stages preceding the given stage and the initial number of offspring, both of which may vary considerably from year to year. Processes occurring at early life stages are thus of great importance as they shape the dynamics of the reproductive life-stage. The question of how stochastic and density-dependent processes affect early life-stages of an organism is therefore of general interest to population ecologists, and has recently attracted increased attention due to our efforts to predict the biological consequences of climate change.

While there generally is consensus that density dependence is a feature of population dynamics for most species [3], [4], it is crucial to understand how it operates and when during the life cycle it occurs. The relative timing of density dependence determines how stochastic variation (e.g. through climate) translates into changes in adult abundance. This is of particular interest in fisheries, because harvested fish stocks often show large fluctuations in recruitment [5]–[7]. While climate is expected to strongly affect survival of eggs and larvae in marine fish [8], [9], density dependence often occurs later in life, particularly among juvenile fish [10], [11]. Compensatory density dependence (‘compensation’), which occurs when population growth is reduced at high abundance, tends to reduce population variability and partially offsets the loss of individuals that are harvested [3], [11], [12]. Compensation is implied in stock-recruitment relationships commonly used in models of fisheries population dynamics (e.g. Beverton-Holt). Density-dependent survival in juveniles modifies the impact of climate factors operating early in life, and leads to lower variability in year-class strength of adults compared to early life-stages [10]. Quantifying density dependence in different phases of the life cycle is thus important for conservation and fisheries management.

Evidence of density-dependent survival generally comes from time-series analysis [4], suggesting a causal relationship between population abundance and survival. Assessing consequences of this process for a population is however difficult without a model that incorporates all life-stages of an organism and thus covers the entire life cycle, including a relationship between spawners and recruitment. Life-cycle models dynamically link the multiple effects of intrinsic and/or extrinsic factors on the different life-stages from one time period to the next, thereby propagating information and uncertainty about influences on one life-stage to inform processes affecting other life-stages [13]. This is crucial for evaluating population-level effects of multiple intrinsic and extrinsic factors that operate at different times during a life cycle, such as density dependence, climate variation, and harvesting.

Here, we develop a life-cycle model for the population dynamics of a large marine fish, Atlantic cod (*Gadus morhua*). The Northeast Arctic (NEA) stock is currently the largest cod stock in the world and sustains a large and profitable fishery. Using a state-space approach we are able to incorporate catch statistics and scientific survey data on all cod life-stages from different time periods (covering >50 years), while accounting for a stochastic environment, inaccurate sampling, and non-independence of data. Our goal is to disentangle the effects of stochasticity and density regulation on the survival of pre-recruit life-stages, and to quantify the strength of density dependence and its consequences for population variability.

## Methods

### Northeast Arctic cod

**Figure 1:**
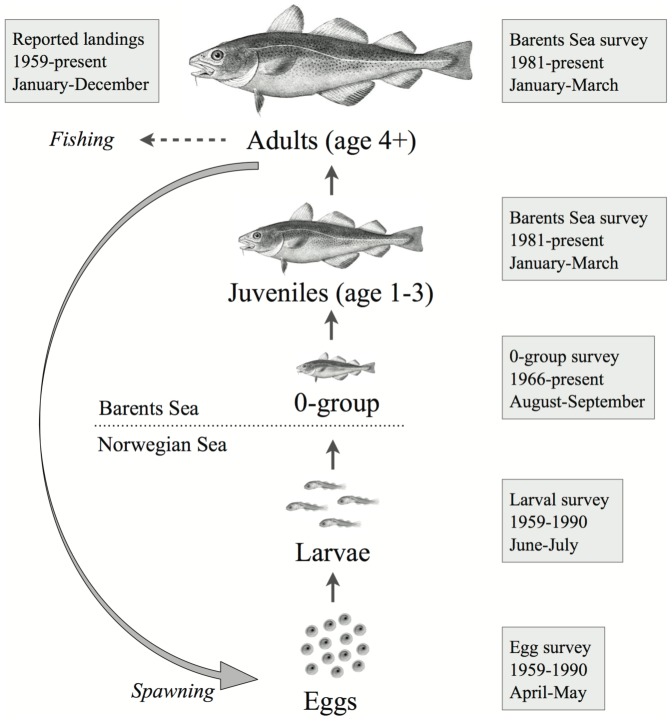
Schematic illustration of the NEA cod life cycle and available observational data.

Northeast Arctic cod are distributed along the coast of Norway and throughout the Barents Sea. Mature cod spawn from February to April along the Norwegian coast, particularly around the Lofoten islands. Spawned eggs drift northeastward along the coast in the Norwegian Coastal Current, and hatch into larvae less than a month after spawning and before reaching the feeding grounds in the Barents Sea. By late summer the 0-group fish (pelagic juveniles) are distributed over large areas of the Barents Sea, where they settle to the bottom in late autumn (demersal juveniles). The fish remain in the Barents Sea until they reach maturity and start their annual migrations to the spawning grounds, typically at 6-8 years old [14]. Fig. 1 shows the general life cycle and ecology of NEA cod and the observational data used in this study. Over the past 65 years, total stock biomass of NEA cod has fluctuated between 1 and 4 million tons, and annual fishery landings of 0.2–1.3 million tons have been reported [15].

### Data sources

We used two types of observational data: indices of age-specific abundances from scientific surveys and age-specific fisheries landings. Fisheries landings data (1959-present) were obtained from international statistics based on NEA cod landings in the Norwegian and Barents Seas [15]. Indices of the abundance of cod eggs and larvae (1959–1990) were obtained from ichthyoplankton surveys in spring (April-May) and summer (June-July) in the Norwegian and Barents Seas by the Knipovich Polar Research Institute of Marine Fisheries and Oceanography (PINRO), Murmansk, Russia [16], [17]. An abundance index for 0-group cod (1966-present) was obtained from autumn samplings (August-September) by the international 0-group survey in the Barents Sea [15], [18] as described in [19]. Abundance indices of juvenile and adult cod of age 1–9 (1981-present) were obtained from Norwegian bottom trawl surveys in winter in the Barents Sea [15]. The above-mentioned surveys have good spatial coverage in those areas inhabited by the different cod life-stages. We used annual data (1959-present) on weight-at-age and age-specific probabilities of being mature [15] to calculate spawning stock biomass from the estimated abundances. Temperature records (1959-present) were obtained from the Kola meridian transect (33°30′E, 70°30′N to 72°30′N), a good indicator of the thermal conditions in the Barents Sea. Monthly values were calculated by averaging temporally along the transect and vertically from 0–200 m water depth (historical data from [20]; recent values provided by PINRO: www.pinro.ru).

### Process model

The process model describes changes in age-specific abundance over time. It consists of a dynamic age-structured population model with lognormal process noise, which refers to stochasticity in the age-/stage-specific survival due to environmental effects. Spawning stock biomass (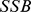) in a given year (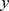) is calculated from age-specific abundance (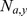), weight-at-age (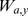), and the probability of being mature (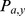):
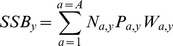



Here, 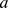 denotes age and 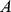 is maximum age. The number of eggs (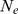) is then calculated from spawning stock biomass and fecundity:
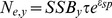



Here, 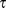 is mean fecundity (eggs kg^−1^), and 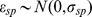 is a Gaussian error term with mean zero and variance 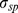 describing stochasticity in the spawning process, that is, process error associated with mean fecundity, fertilization of eggs, and egg survival prior to the egg survey (for estimates of annual egg production see [21]). The number of larvae (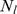) is calculated from egg abundance and mortality (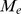, day^−1^) during the period between egg and larval surveys (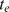, days):
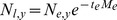



Previous studies have shown that warmer temperature is associated with higher survival of early life-stages of cod, particularly larvae [22], [23]. We tested for temperature dependence of egg and larval survival, and found that the effect on egg survival was highly correlated with and weaker than the effect on larval survival. We thus model temperature effects on larval but not egg survival. The number of 0-group fish (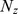) is calculated from larval abundance and mortality (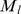, day^−1^) during the period between larval and 0-group survey (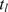, days), and a temperature effect (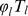) based on a proxy of the thermal conditions during the larval stage (June-August):
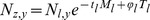



It is worth noting that during the period between the larval and 0-group surveys most of the larvae undergo metamorphosis after which they are considered ‘post-larvae’ or ‘early juveniles’. For simplicity, we refer to the mortality experienced during this period as the ‘larval mortality’.

**Figure 2:**
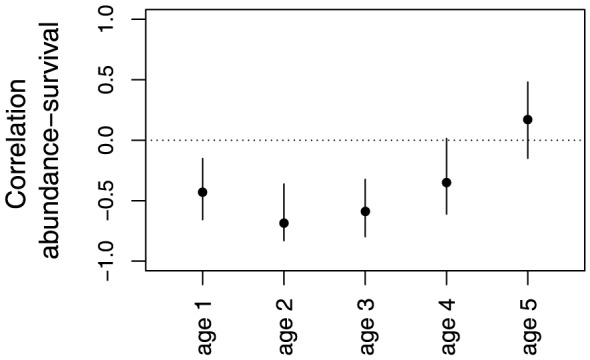
Correlations between log-abundances and survival indices for cod age-classes 1–5. The survival index was based on observational data and calculated as the difference between the log numbers of the subsequent and previous age-classes. Observation errors for the survival and abundance indices are thus independent and the correlation is not biased due to correlated errors. Circles and error bars indicate medians and 95% confidence intervals from bootstrapped samples, which were constructed by randomly sampling from the available abundance-survival pairs with replacement (repeated 10000 times).


We considered density-dependent survival among age-classes 0–3. Intercohort predation (i.e. cannibalism) from older cod is known to decrease the survival of juveniles [15]. Because mortality due to cannibalism is expected to be particularly strong at the 0-group stage after the cod have entered the Barents Sea and settled to the bottom [24], we modeled cannibalism between the 0-group and cannibalistic cod of age 3–4. This choice was based on a correlation analysis between age-class abundances and 0-group process error using a model without cannibalism. Furthermore, intracohort density dependence was modeled for ages 1–3 based on an initial analysis of the observational data, which indicated density-dependent survival within these age-classes (Fig. 2). This analysis consisted of calculating the correlation between age-class abundance and a survival index, which was derived from the abundances of the same cohort the previous and subsequent years. This method ensures that the correlation is not biased due to correlated errors, as the estimation errors for the survival and abundance indices are independent [10]. Furthermore, based on a preliminary test of the form of density dependence, we used a Beverton-Holt relationship (see Text S1 in File S1), that is, we model compensation in survival, but no overcompensation (as implied by the Ricker model). The number of age 1 fish (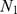) is thus calculated from 0-group abundance the previous year, an intercohort interaction (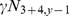) describing the increase in mortality with abundance of older cannibalistic cod, and mortality (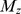, day^−1^) during the period between 0-group and age 1 survey (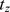, days):

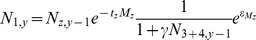
, where 
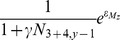
 was restricted to be <1.

Here, the term 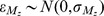 accounts for variability in survival of the 0-group. Accordingly, the number of age 2–4 fish is calculated from the abundance of the previous age-class in the previous year, juvenile mortality (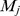), and an intracohort interaction (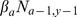) describing the increase in mortality with abundance:


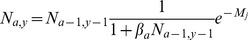
, for 2≤a≤4

In our model, cod are recruited to the fishery at age 4, and fishing mortality on age 3 is assumed to be negligible [15]. The number of age 5–9 fish (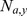) is thus calculated from the abundance of the previous age-class in the previous year, natural mortality of adults (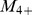), and fishing mortality on that age-class the year before (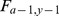):


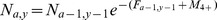
, for 5≤a≤9

This formulation assumes that natural and fishing mortality both occur throughout the year. Fishing mortality for a given age and year is modeled based on the age-selectivity of the fishery (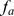), and a year effect to account for temporal changes in fishing effort (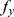):
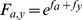



Here, 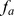 represents the mean relative proportion by age of fish caught in the fishery, and 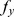 is a measure of fishing effort that is unknown and estimated in the model [25]. Separable year and age effects imply that total effort may have changed over time, but that no systematic changes in the age-selectivity of the fishery have occurred during the time period considered here. We allowed for effort to change gradually over time due to changes in fisher's behavior, investments or regulations [25]. It was modeled as a random walk process following a normal distribution according to 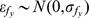:
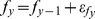



For identifiability, effort in the first year was set to 0 [25], [26]. Using an additional error term 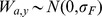 to account for stochastic variation in fishing mortality by age and year did not qualitatively change our results, but significantly increased computation time, and was thus not included in the model (see Text S1 in File S1). Finally, the catch for a given age and year (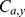) is related to age-specific abundances through:
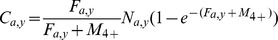



This equation (Baranov catch equation, see [27]) defines the true catch as the fraction of the total removal of individuals from the population due to fishing.

### Observation model

The observation model links the true numbers to the observations. The true age-specific *abundances* and *catches* were treated as latent (unobserved) variables and were related to the survey *indices* and reported *landings* using lognormal errors. Errors in reported landings (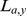) were assumed to be log-normally distributed and independent between ages and years:
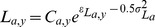



Here, 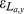 is a Gaussian error term with mean zero and variance 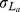, and the term 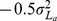 adjusts the mean of the catch error to one for any value of 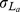 [27]. For the abundance indices, such a correction term was not explicitly included as it only affects the values of the age-specific catchability estimates [25]. Furthermore, the indices of age-specific abundance from the scientific surveys were assumed to be associated with observation error due to the inaccuracy of the scientific sampling. Abundance indices from the Barents Sea bottom trawl survey in winter (age-classes 1–9) were modeled according to:
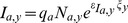



Here, 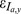 is a Gaussian error term with mean zero and variance 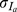, and 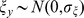 is a random year effect that accounts for correlated observation errors within years [28], [29]. Hence, we estimate one variance parameter (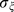) in addition to the nine age-specific observation error variances (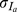). The 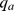 are proportionality constants reflecting the age-specific catchability of the survey. Because in 1993/1994 the sampling area of the Barents Sea bottom trawl survey and the mesh size in the codend were changed [30], survey data for ages 3+ were corrected according to [31]. Since no correction factors were available for ages 1 and 2, the catchability of these age-classes was modeled independently for the two periods before (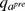) and after (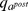) 1993.

Because eggs, larvae, and the 0-group were sampled by different surveys, we used stage-specific error terms (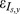) and proportionality constants (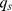) for each of these three life-stages:
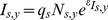



Thus, the constants 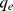, 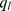 and 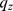 are the catchabilities, and 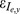, 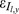, and 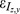 are the Gaussian error terms with variances 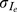, 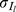, and 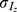 for the egg, larval and 0-group indices, respectively.

### Bayesian parameter estimation

We use a Bayesian framework for estimating the model parameters, which facilitates the incorporation of prior knowledge about parameter values. The priors are updated by the likelihood functions of the model to estimate the joint posterior distribution of all parameters given the observations. The use of this framework allows for the quantification of uncertainty in the parameter estimates and the model predictions. The Bayesian analysis was performed using JAGS [32], a program that uses Gibbs sampling [33], a variety of Markov Chain Monte Carlo (MCMC), to estimate the joint posterior distribution of the model parameters. We ran JAGS from within R (ver. 3.0.2, [34]) using the package R2jags [35]. MCMC runs consisted of three chains with a burn-in of 250 000 samples, and a posterior distribution based on 250 000 samples for each chain. We used a thinning of 1000 to reduce autocorrelation in the chains. This gave a total of 750 samples per parameter for the posterior distributions.

### Parameter values and prior distributions

We fix the values for some of the parameters for which independent estimates were available, specifically mean fecundity and natural mortality of eggs and larvae. We used the following values: 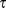  = 235 eggs g^−1^ for mean fecundity [21], 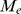  = 0.169 day^−1^ for egg mortality, and 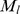  = 0.058 day^−1^ for larval mortality [36], [37].

Mortality during the period from larval to 0-group survey was calculated assuming 60 days of larval stage (0.075 day^−1^) and 60 days of early juvenile stage (0.04 day^−1^), thus accounting for differences in mortality before and after metamorphosis [36], [37]. We used informative priors for the density-independent natural mortality of the 0-group (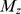) and the demersal juveniles (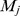) (year^−1^), which were set to be log-normally distributed with medians 0.425 [36] and 0.2 [38], [39], and a variance of 0.5. Since the relative differences in catchability of the survey are not known, estimates of total mortality in these ages will be confounded with the catchability estimates [25]. We thus used informative priors to restrict the parameter estimates to a biologically reasonable range, and tested the sensitivity of our results to this assumption (see Supporting Information). Natural mortality of adults was set to 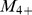 = 0.2 year^−1^ following [39]. This value was set in order to achieve model convergence, as is commonly done in non-Bayesian and VPA-type analyses [15]. Although a fixed natural mortality of adults poses a general limitation, our approach was developed to investigate mechanisms operating at the early life-stages before the fish are recruited to the fishery (see [25] on estimating temporal trends in natural mortality). Vague priors were used for the age-specific (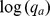) and the 0-group (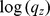) log-catchabilities [25], which were set to be normally distributed with variance 1000 and means 0. The log-catchabilities of eggs (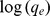) and larvae (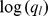) were given normal priors with variance 1000 and means −25. The age-specific fishing selectivities (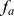) were given normal priors with variance 1000 and means −1.6, and restricted to negative values. These mean values of the vague priors reflect informed yet arbitrary choices, and the posterior distributions do not depend upon them. Uniform priors [0, upper limit] were used for the density dependence parameters (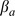), and the observation and process errors (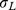, 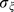, 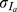, 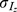, 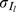, 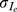, 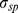, 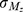, 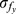). The upper limits of uniform priors were selected such that they were not approached during the sampling process.

### Model diagnostics

We assessed model convergence using common diagnostics [40]–[42], which confirmed convergence of our model with the number of samples used for the posterior distributions. In addition to these formal diagnostics we used informal criteria to ensure model convergence: chains were initialized with different starting values to address problems related to meta-stability in parameter space, trace and density plots were used to detect improper mixing and asymptotic behavior of chains (Fig. S1 in File S1). Furthermore, autocorrelation in the chains was consistently below 0.2 for all parameters (Fig. S2 in File S1), and the posterior samples generally showed low cross-correlations, with the notable exception of the age-effects in fishing mortality (Fig. S3 in File S1).

## Results

The variation associated with the spawning process (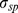) and 0-group survival (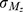) exemplifies the strong impact of environmental stochasticity on the cod population dynamics (Fig. S4 in File S1). A process error standard deviation of 0.7 roughly corresponds to a 2-fold variation in egg abundance. Furthermore, higher environmental temperatures were associated with higher larval survival (but note that the 95% credible interval includes the zero value). Based on the median value of the posterior distribution, mean survival over the entire period from larval to 0-group survey varied between 0.08% and 0.12% depending on temperature during larval development. While these numbers seem small in absolute terms, they imply a 1.5-fold increase in the number of surviving larvae between the coldest and warmest years. We did not include a temperature effect on egg survival, because this parameter was estimated to be close to zero and was highly correlated with the effect on larval survival.

**Figure 3:**
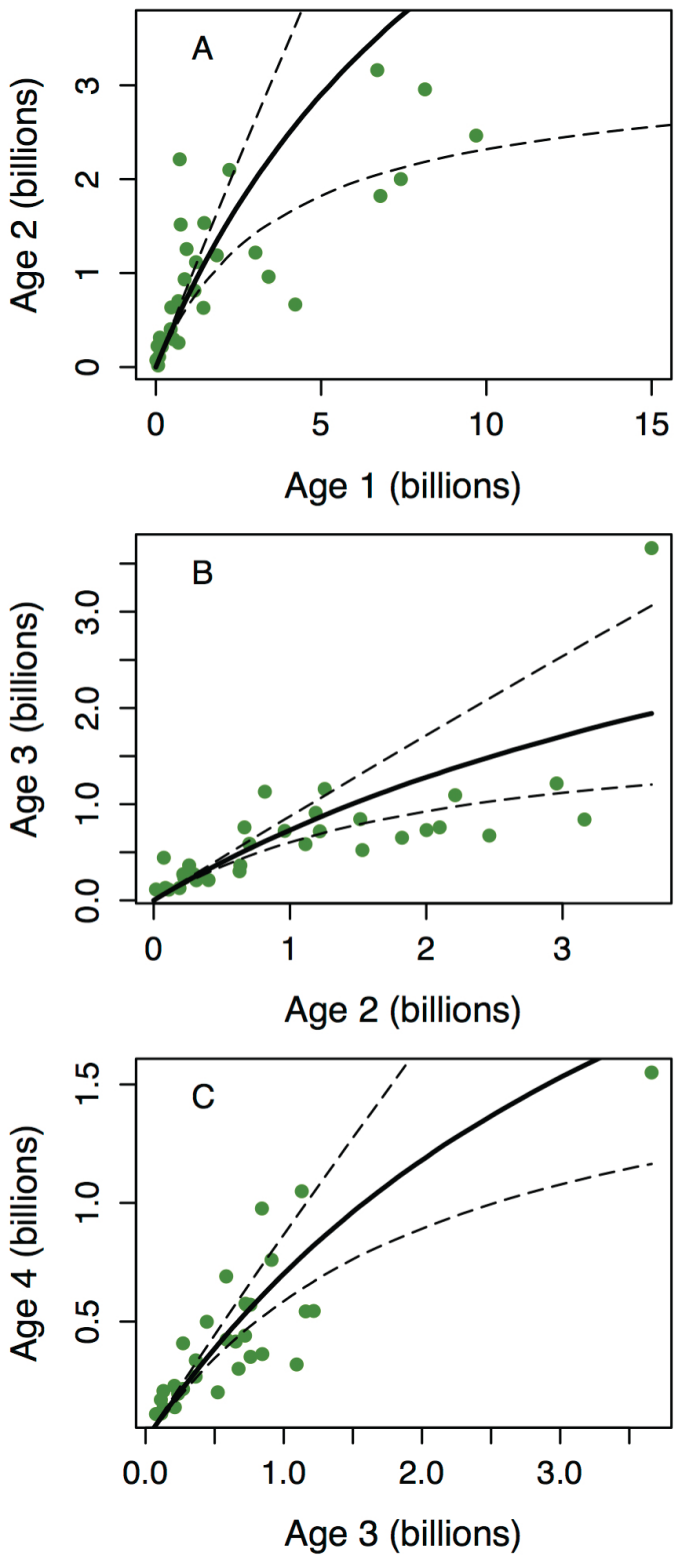
Relationships between cod age-class abundances. Observations (circles) were corrected for estimated survey catchability. Model predictions are shown as medians (solid lines) and 95% credible intervals (dashed lines), which were obtained by sampling parameter values independently for each MCMC draw to account for pairwise correlations between parameters.

The density-independent natural mortality rates of the 0-group fish (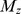 = 0.35 year^−1^), and older juveniles (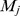 = 0.15 year^−1^), had median estimates that were similar but not identical to the median values of the prior distributions. The effect of cannibalistic older cod (ages 3–4) on the survival of the 0-group was clearly estimated (median [95% credible interval]: 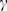 = 1.82^−9^ [7.27e^−10^–3.28e^−9^]), and intracohort density-dependent survival was detected in juveniles of age 1 to 3 (medians [95% credible intervals]: 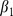 = 9.48^−11^ [5.37e^−12^–2.69e^−10^], 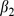 = 1.69^−10^ [8.12e^−12^–4.26e^−10^], 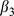 = 2.27^−10^ [1.56e^−11^–4.75e^−10^]). The posterior distributions show that an intracohort density term was well estimated for ages 1, 2, and 3 (Fig. S5 in File S1). The resulting overall mean survival rates of ages 1–3 were 0.77, 0.75, and 0.76, respectively, while the mean 0-group survival was 0.41. Using the estimates of the density-dependent and density-independent survival rates, we can predict the relationships between subsequent juvenile age-classes and compare them to the observed data (Fig. 3). This validation shows that the functional form of density dependence (Beverton-Holt) predicts the relationship between age-class abundances well, and that the model estimates accurately reflect the uncertainty in these relationships.

**Figure 4:**
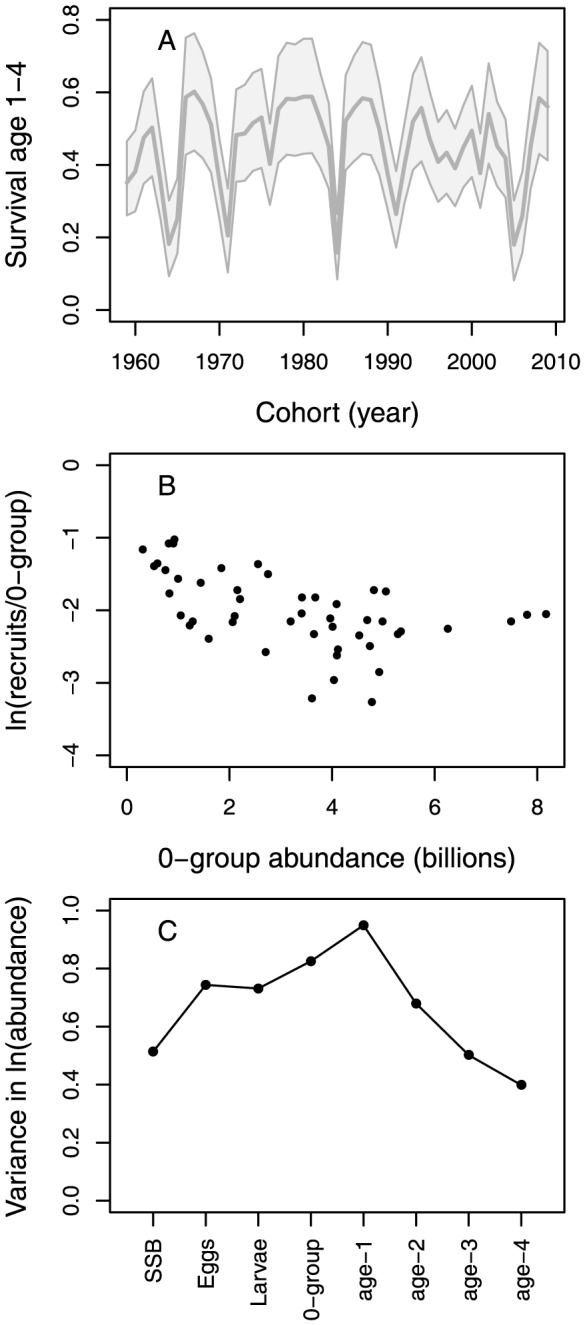
Juvenile survival rate and variance in life-stage or age-class specific abundance. Shown are (A) survival rate from age 1 to 4, (B) relationship between the number of recruits per 0-group and 0-group abundance, and (C) interannual variance of the log-abundance time-series. Lines in (A) represents median values (thick) and 95% credible intervals (thin), obtained by calculating survival from age 1 to 4 independently for each MCMC draw in order to account for parameter correlations. In (C) variance is shown for log-transformed estimates of spawning stock biomass (SSB) and abundances of the early life-stages and age-classes 1–4.

The strength of density dependence in juvenile survival is evidenced by the estimated time series of overall survival from age 1 to age 4, which depends on the combined intracohort density-dependent mortality rates in addition to the density-independent mortality (Fig. 4A). The number of recruits (age 4) per 0-group cod decreases with increasing abundance (Fig. 4B). To further quantify the degree of density dependence among juveniles and its dampening effect on the variability in year-class strength, we calculated the ratio between the largest and smallest year-classes before and after intracohort density dependence at age 1 and age 4. The difference between the largest and smallest year-class is more than 43-fold at age 1 and about 11-fold at age 4. Overall, the inter-annual variance in the estimated time series increases from about 0.51 in SSB to 0.95 at age 1 and thereafter decreases again to 0.40 at age 4 (Fig. 4C).

**Figure 5:**
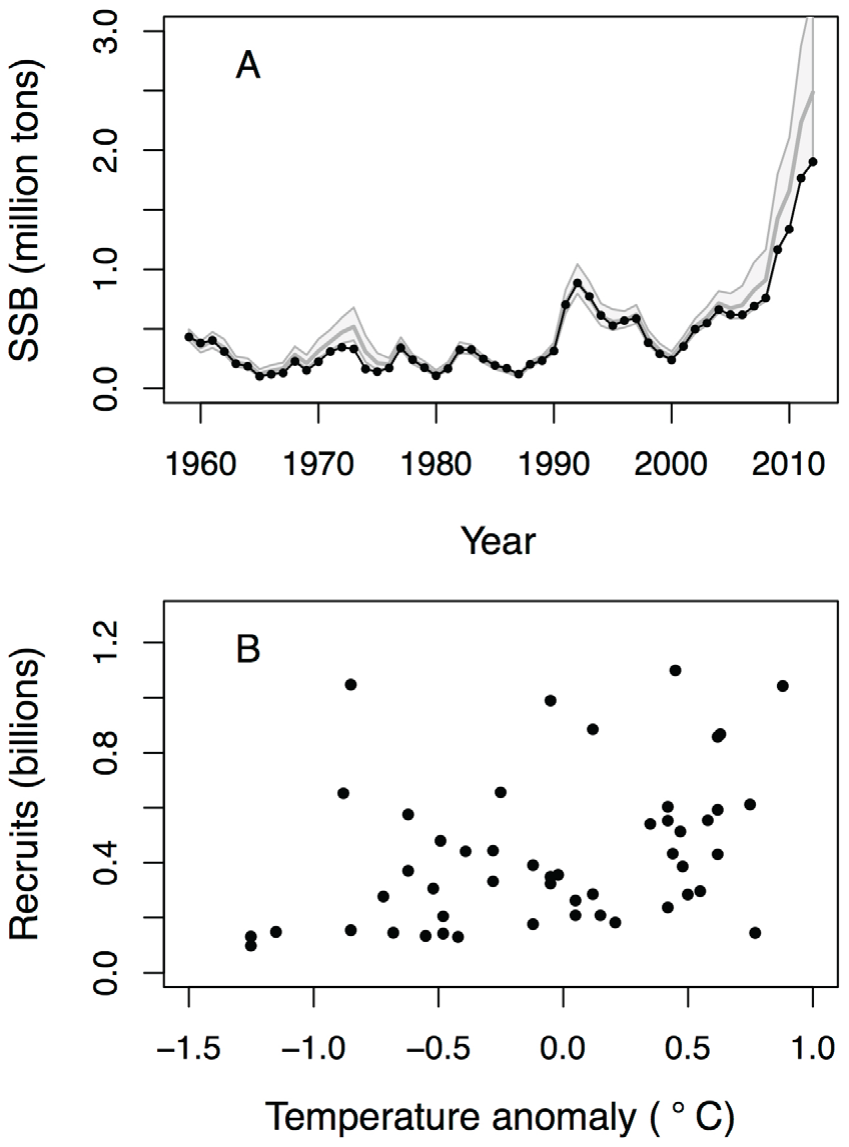
Temporal trend in spawning stock biomass and temperature-recruit relationship. (A) Predicted spawning stock biomass (grey lines, median values with 95% credible intervals) in comparison with the VPA assessment (black line and circles, ICES 2013). (B) Relationship between predicted number of recruits (age 4) and temperature anomaly during the larval stage.

The age-specific fishing mortality shows a smooth hump-shaped relationship, with the lowest average mortality at age 4 and the highest average mortality at age 8–9 (Fig. S6 in File S1). The year-effect in fishing mortality, which represents gradual changes in effort over time, shows a temporal trend that reflects the historic changes in fishing pressure (Fig. S7 in File S1). Fishing mortality slightly increased until the late 1980s, then dropped significantly within a few years, returned to previous levels in the late 1990s, and has been declining continuously ever since. The resulting temporal dynamics in spawning biomass as predicted by the model (Fig. 5A) are in close correspondence with estimates from a virtual population analysis [15]. This is not surprising given that the same data and natural mortality of adults (age 4+) were used in our model. Abundance trends for the different life-stages and age-classes as predicted by the model are further in general agreement with the temporal dynamics seen in the observational data (Fig. S8 in File S1). The age-specific observation errors show a U-shaped relationship, with the lowest observation error on 6-year-old cod (Fig. S9 in File S1) and the highest observation error on the 0-group. The catchability estimates for the survey suggest that surveyability of age-classes 1–2 increased after 1993, and that surveyability continuously declines from age 3 to 9 (Fig. S10 in File S1). All parameter estimates are presented in Table S1 in File S1.

## Discussion

We studied the impacts of density-independent and density-dependent factors on early life-stage survival and the consequences of these processes for the variability in adult year-class strength. We did this by developing a dynamic population model for NEA cod based on catch statistics and scientific survey data for all life-stages, from eggs to spawners, while accounting for the inaccuracy of the observations and stochasticity in the population dynamics. The parameter estimates highlight the importance of density dependence for population regulation. Intracohort density dependence in survival occurs among juveniles of age 1–3, indicating that this period of the life cycle determines the compensatory capacity of the stock. This compensation dampens the variation in early life-stage abundances (egg, larvae, and 0-group) and thus reduces the impact of environmental stochasticity on the recruitment to the fishery.

Environmental stochasticity has a strong impact on the production of eggs and the survival of larvae and 0-group cod. First, egg production as well as 0-group survival are highly stochastic processes as evidenced by the large variances of the process errors. Second, temperature has a positive effect on the survival of larval cod, which is in line with other studies suggesting that growth and survival of cod early life-stages depend on temperature [22], [23]. Temperature-dependent survival may be caused by direct and/or indirect effects. Higher temperatures may either directly increase survival, for instance through faster development, which reduces the duration and thus total mortality of a life-stage, or indirectly increase survival through an increase in food supply. Interestingly, low temperatures consistently result in low recruit abundance, whereas high temperatures may result in either small, medium or large year-classes (Fig. 5), suggesting that warm temperature is a necessary but not a sufficient condition for good recruitment [43].

Density dependence in the juvenile life-stage was found to be compensatory, that is, increased densities reduce the survival of the juvenile age-classes. Although density-dependent processes can be compensatory or depensatory, previous studies have suggested compensatory dynamics in NEA cod [10], [36], whereas depensation does not seem to occur in this stock within the range of historically observed abundances [44]. Recently, it was shown that the variance in cohort abundance of NEA cod increases up to age 1 and subsequently decreases until age 3, indicating a dampening effect of density dependence on cohort fluctuations [37]. Another study found evidence for density dependence at ages 1–3 by correlating estimates of age-specific process errors (using a similar approach as adopted here) to the corresponding abundances [38].

Both competition and cannibalism from older cod have previously been suggested as a cause of density-dependent survival of juvenile cod [24], [45]–[48]. In this study, cannibalism from older cod was found to affect the survival of 0-group cod, while intracohort density dependence was found to be a significantly better predictor of survival among age 1–3 juveniles than cannibalism (intra- and intercohort terms for a given age-class could not be estimated at the same time). Cannibalism is expected to affect juvenile survival after the cod have entered the Barents Sea and settled to the seafloor. In fact, the most abundant cod length group in the stomachs of larger cannibalistic cod is about 10–14 cm [24], which roughly corresponds to the 0-group stage. During the period from the 0-group survey in August-September to the bottom-trawl survey in January-March the cod grow from ∼6–9 cm [22] to ∼12–15 cm [49]. Competition is also expected to become significant when the cod settle to the seabed and assume a demersal existence. However, it has also been reported that older juveniles (ages 2–3) are more concentrated in the southeastern Barents Sea, thereby indicating higher densities [14]. In line with these observations, our results suggest that density dependence occurs in ages 1 to 3. Separating this effect by age group is difficult due to pairwise correlations among parameter estimates. However, as illustrated by Fig. 4, overall juvenile survival is clearly reduced at high cohort densities. On the other hand, we were not able to estimate an intracohort density dependence term for the 0-group, possibly due to the high observation error of these data [17], [19], which is reflected in our estimated variance of the 0-group observation error (Table S1 in File S1). In summary, both cannibalism and competition affect the survival of juveniles. Importantly, the intracohort density dependence among juveniles of age 1–3 considerably dampens the variability in cohort abundance.

The relatively strong compensation within the juvenile life-stage dampens the large variation in abundance of the early life-stages caused by environmental stochasticity. The condition of the spawning stock, here included through the weight-at-age and probability of being mature, as well as climatic and oceanographic conditions during the spawning period presumably have a strong effect on egg production. In addition, temperature is associated with changes in survival of cod larvae. Due to the density regulation in the demersal juvenile stage (ages 1–3), the variation in the abundance of cod entering the fishery is considerably lower than for the preceding age-classes, and similar to levels of recruitment variation that are commonly observed for marine fishes [7]. Higher abundances at the larval and post-larval stage (0-group) imply lower juvenile survival due to density-dependent survival, which operates after the stochastic impacts on the spawning process and early life-stages. The negative slope of the relationship between survival rate from 0-group to recruitment at age 4 and abundance reflects the degree of compensation in this population (Fig. 4B). Consequently, one implication of density-dependent survival is that it reduces the impact of climate variability on the year-class strength of recruits. The fishery is thus less affected by climate than would otherwise be expected in the absence of density regulation in the juvenile life-stage. It is further known that recruitment is positively linked to spawner abundance in NEA cod [50], [51], and that the incidence of poor recruitment increases at spawner biomasses below about half a million tons [52]. In this study, we assumed a linear relationship between spawner biomass and egg production with a lognormal error term. With its current total stock biomass of ∼3.5 million tons, the NEA cod continues to sustain an enormous fishery with recent annual catches around 1 million tons.

In conclusion, the timing of stochastic versus regulatory events plays a crucial role in determining adult abundance in marine fish populations. Using a life-cycle model for NEA cod that dynamically links the population life-stages, we have demonstrated that environmental stochasticity, including climatic variation, affects the survival of early life-stages of cod, while density-dependent survival occurs at the juvenile stage (age 1–3). Compensatory density dependence at the juvenile stage reduces the impact of climatic and other stochastic processes operating earlier in life, as well as changes in spawning stock biomass due to harvesting, thus acting to stabilize temporal variation in the abundance of older life-stages. This type of density regulation in demographic rates is the basis of sustainable harvest as it causes population growth to increase at low abundance, and is therefore fundamental to population persistence.

## Supporting Information

File S1
**Contains the files: Text S1: Model tests and extensions.** **Table S1: Posterior medians and 95% credible intervals for all model parameters.** **Figure S1: Posterior distributions of all model parameters.** **Figure S2: Temporal autocorrelation of all model parameters.** **Figure S3: Cross-correlations of all model parameters. Figure S4: Estimated process errors.** Shown are the process errors for spawning (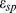) and early juvenile mortality (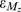). **Figure S5: Prior and posterior distributions of mortality rates.** Prior (grey) and posterior (black) distributions are shown for the density-independent mortality of the 0-group (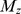) and ages 1–3 (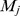) and posterior distributions (uniform priors) are shown for the density-dependent mortality rates of ages 1–3 (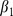, 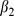, 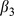). **Figure S6: Parameter estimates for the age-specific fishing mortality.** **Figure S7: Estimated year-effect of the fishing mortality.** **Figure S8: Abundance trends for eggs, larvae, 0-group cod, and age-classes 1–9.** Shown are the observations (black), corrected for age-specific catchability, and the model predictions (grey) with 95% credible intervals. Note the log-scale and the different periods for the abundance time-series corresponding to the actual observations (eggs/larvae: 1959–1990; 0-group: 1966–2010; ages 1–9: 1981–2010). **Figure S9: Estimates of age-specific observation errors of the Barents Sea survey.** **Figure S10: Estimates of age-specific catchabilities of the Barents Sea survey.** For age-classes 1 and 2 surveyability was independently estimated for the period before 1993 (open circles).(ZIP)
