# Supplementary material for: Stochasticity and Determinism: How Density-Independent and Density-Dependent Processes Affect Population Variability
Source: PLoS One. 2014 Jun 3;9(6):e98940. doi: 10.1371/journal.pone.0098940 (PMC4044037; doi:10.1371/journal.pone.0098940)
Supplement: File S1 — Contains the files: Text S1: Model tests and extensions. Table S1: Posterior medians and 95% credible intervals for all model parameters. Figure S1: Posterior distributions of all model parameters. Figure S2: Temporal autocorrelation of all model parameters. Figure S3: Cross-correlations of all model parameters. Figure S4: Estimated process errors. Shown are the process errors for spawning () and early juvenile mortality (). Figure S5: Prior and posterior distributions of mortality rates. Prior (grey) and posterior (black) distributions are shown for the density-independent mortality of the 0-group () and ages 1–3 () and posterior distributions (uniform priors) are shown for the density-dependent mortality rates of ages 1–3 (, , ). Figure S6: Parameter estimates for the age-specific fishing mortality. Figure S7: Estimated year-effect of the fishing mortality. Figure S8: Abundance trends for eggs, larvae, 0-group cod, and age-classes 1–9. Shown are the observations (black), corrected for age-specific catchability, and the model predictions (grey) with 95% credible intervals. Note the log-scale and the different periods for the abundance time-series corresponding to the actual observations (eggs/larvae: 1959–1990; 0-group: 1966–2010; ages 1–9: 1981–2010). Figure S9: Estimates of age-specific observation errors of the Barents Sea survey. Figure S10: Estimates of age-specific catchabilities of the Barents Sea survey. For age-classes 1 and 2 surveyability was independently estimated for the period before 1993 (open circles). (ZIP) [file pone.0098940.s001.zip › Ohlberger et al Supporting Information/Table S1.pdf]

Table S1. Posterior medians and 95% credible intervals for all model parameters

Symbol	Median	95% Credible Interval		Life-stage/age-class
Density-independent mortality				
M_z	0.341	0.146	0.804	0-group
M_j	0.143	0.065	0.252	Ages 1-3
Density-dependent mortality (intracohort)				
β_1	9.48e-11	5.37e-12	2.69e-10	Age 1
β_2	1.69e-10	8.12e-12	4.26e-10	Age 2
β_3	2.27e-10	1.56e-11	4.75e-10	Age 3
Density-dependent mortality (intercohort)				
γ	1.82e-9	7.27e-10	3.28e-9	0-group
Temperature effect				
φ_l	0.150	-0.242	0.562	Larvae
Process errors				
σ_{sp}	0.731	0.522	0.986	Spawning
σ_{M_z}	0.778	0.479	1.194	0-group
Survey catchability (surveyability)				
q_e	4.56e-13	2.94e-13	6.55e-13	Eggs

q_l	5.31e-14	3.60e-14	7.96e-14	Larvae
q_z	4.948	2.853	8.922	0-group
$q_{1^{pre}}$	0.068	0.027	0.170	Age 1 pre 1993
$q_{2^{pre}}$	0.181	0.099	0.360	Age 2 pre 1993
q_1	0.722	0.326	1.556	Age 1
q_2	0.351	0.201	0.603	Age 2
q_3	0.327	0.244	0.428	Age 3
q_4	0.320	0.282	0.363	Age 4
q_5	0.292	0.259	0.330	Age 5
q_6	0.252	0.227	0.279	Age 6
q_7	0.195	0.173	0.220	Age 7
q_8	0.120	0.101	0.146	Age 8
q_9	0.074	0.056	0.096	Age 9
Fishing mortality year-effect				
σ_{f_y}	0.223	0.174	0.280	-
Fishing mortality age-effects				
f_4	-1.788	-2.032	-1.570	Age 4
f_5	-1.079	-1.262	-0.888	Age 5

f_6	-0.745	-0.933	-0.552	Age 6
f_7	-0.521	-0.727	-0.339	Age 7
f_8	-0.400	-0.603	-0.218	Age 8
f_9	-0.394	-0.623	-0.195	Age 9
f_{10}	-0.490	-0.806	-0.181	Age 10
f_{11}	-0.683	-1.190	-0.142	Age 11
Observation errors abundance indices				
σ_{I_e}	0.810	0.569	1.125	Eggs
σ_{I_l}	0.763	0.514	1.122	Larvae
σ_{I_z}	1.820	1.435	2.305	0-group
σ_{I_1}	1.527	1.213	1.897	Age 1
σ_{I_2}	1.045	0.822	1.371	Age 2
σ_{I_3}	0.550	0.419	0.759	Age 3
σ_{I_4}	0.249	0.183	0.358	Age 4
σ_{I_5}	0.191	0.131	0.267	Age 5
σ_{I_6}	0.107	0.023	0.184	Age 6
σ_{I_7}	0.177	0.118	0.260	Age 7

σ_{I_8}	0.432	0.324	0.588	Age 8
σ_{I_9}	0.688	0.543	0.930	Age 9
Observation error correlation within years (abundance indices)				
σ_{ξ}	0.214	0.149	0.300	Ages 1-9
Observation errors reported landings				
σ_{L_4}	0.550	0.452	0.700	Age 4
σ_{L_5}	0.345	0.276	0.447	Age 5
σ_{L_6}	0.224	0.168	0.304	Age 6
σ_{L_7}	0.253	0.203	0.327	Age 7
σ_{L_8}	0.205	0.162	0.262	Age 8
σ_{L_9}	0.188	0.139	0.251	Age 9
$\sigma_{L_{10}}$	0.311	0.244	0.400	Age 10
$\sigma_{L_{11}}$	0.454	0.362	0.583	Age 11
