# Supplementary material for: Stochasticity and Determinism: How Density-Independent and Density-Dependent Processes Affect Population Variability
Source: PLoS One. 2014 Jun 3;9(6):e98940. doi: 10.1371/journal.pone.0098940 (PMC4044037; doi:10.1371/journal.pone.0098940)
Supplement: File S1 — Contains the files: Text S1: Model tests and extensions. Table S1: Posterior medians and 95% credible intervals for all model parameters. Figure S1: Posterior distributions of all model parameters. Figure S2: Temporal autocorrelation of all model parameters. Figure S3: Cross-correlations of all model parameters. Figure S4: Estimated process errors. Shown are the process errors for spawning () and early juvenile mortality (). Figure S5: Prior and posterior distributions of mortality rates. Prior (grey) and posterior (black) distributions are shown for the density-independent mortality of the 0-group () and ages 1–3 () and posterior distributions (uniform priors) are shown for the density-dependent mortality rates of ages 1–3 (, , ). Figure S6: Parameter estimates for the age-specific fishing mortality. Figure S7: Estimated year-effect of the fishing mortality. Figure S8: Abundance trends for eggs, larvae, 0-group cod, and age-classes 1–9. Shown are the observations (black), corrected for age-specific catchability, and the model predictions (grey) with 95% credible intervals. Note the log-scale and the different periods for the abundance time-series corresponding to the actual observations (eggs/larvae: 1959–1990; 0-group: 1966–2010; ages 1–9: 1981–2010). Figure S9: Estimates of age-specific observation errors of the Barents Sea survey. Figure S10: Estimates of age-specific catchabilities of the Barents Sea survey. For age-classes 1 and 2 surveyability was independently estimated for the period before 1993 (open circles). (ZIP) [file pone.0098940.s001.zip › Ohlberger et al Supporting Information/Text S1.pdf]

MODEL TESTS AND EXTENSIONS

Flexible density dependence function

We tested the model with a flexible density dependence function for juvenile survival according to the Deriso-Schnute model as described in Maunder and Deriso (2011):

$$N_{a,y} = N_{a-1,y-1} e^{-M_a} (1 - \beta_a \vartheta N_{a-1,y-1})^{\frac{1}{\vartheta}}$$

This model uses an additional parameter (ϑ) that can be set to represent the Beverton-Holt ($\vartheta = -1$) and Ricker ($\vartheta \to 0$) models. The ϑ parameters were estimated close to -1 for all juvenile age-classes. We therefore used a Beverton-Holt relationship in our model.

Fishing mortality process error

We further tested the model with an additional process error on fishing mortality to account for stochastic variation in fishing mortality by age and year (Aanes et al. 2007):

$$F_{a,y} = e^{f_a + f_y + W_{a,y}}, \text{ with } W_{a,y} \sim N(0, \sigma_F)$$

This process error can be estimated, but is computationally expensive, and only marginally affects the posterior distributions of the other parameters such as for the mortality terms in juveniles. We therefore did not include this additional error term into our model.

Density-independent mortality priors

We evaluated our model estimates for the density-dependent mortality rates of ages 1-3 (β_a) using different priors for the median and variance of the density-independent mortality rate (M_j). Using median values of 0.1 or 0.3 or variances of 0.2 or 0.8 for M_j resulted in similar β_a estimates as those reported in Table S1 (based on a median of 0.2 and a variance of 0.5). In all cases, the difference between median estimates for all density terms was less than 33%.
