# Supplementary figures and images for: Stochasticity and Determinism: How Density-Independent and Density-Dependent Processes Affect Population Variability
Source: PLoS One. 2014 Jun 3;9(6):e98940. doi: 10.1371/journal.pone.0098940 (PMC4044037; doi:10.1371/journal.pone.0098940)

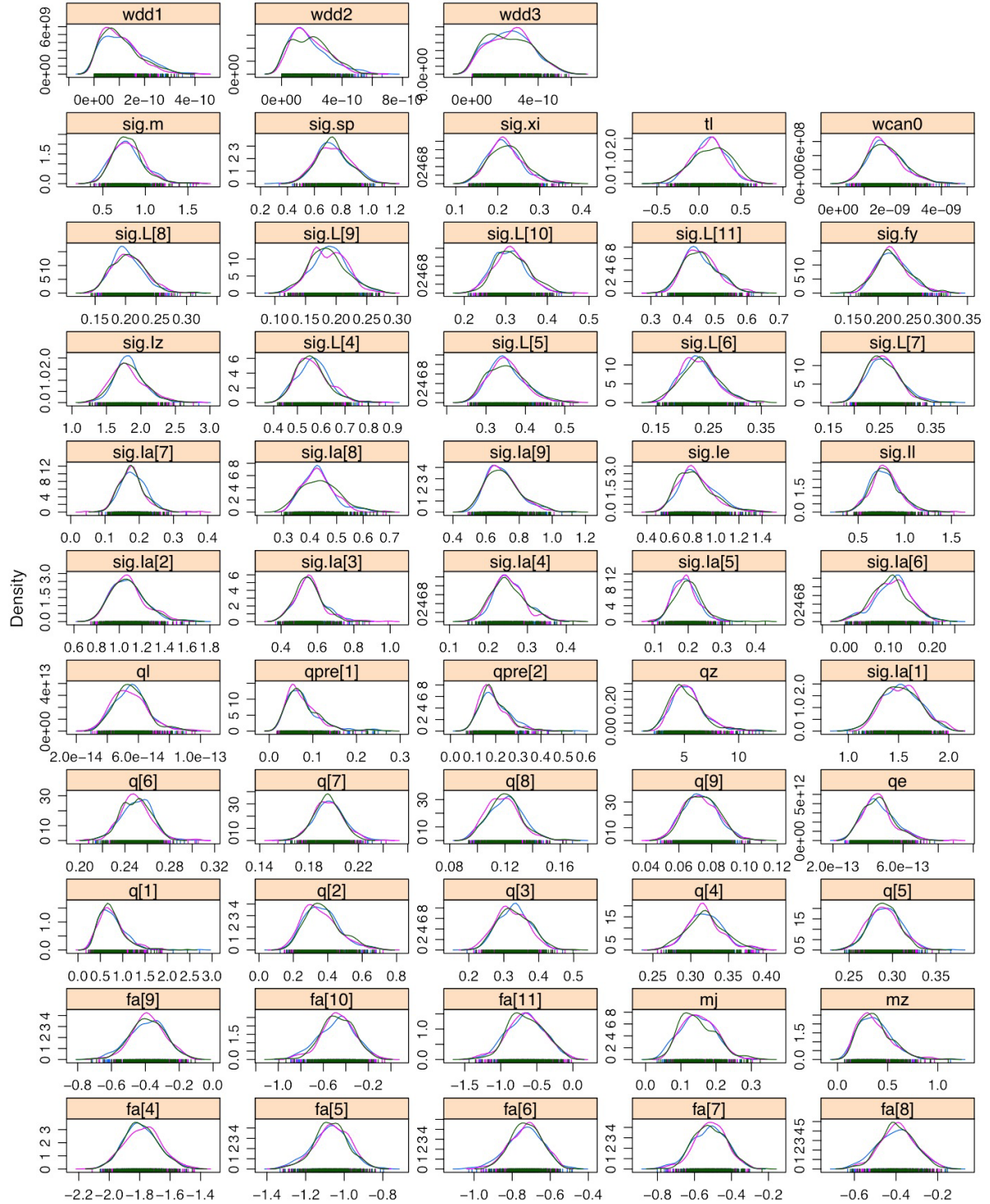

Supplement: File S1 — Contains the files: Text S1: Model tests and extensions. Table S1: Posterior medians and 95% credible intervals for all model parameters. Figure S1: Posterior distributions of all model parameters. Figure S2: Temporal autocorrelation of all model parameters. Figure S3: Cross-correlations of all model parameters. Figure S4: Estimated process errors. Shown are the process errors for spawning () and early juvenile mortality (). Figure S5: Prior and posterior distributions of mortality rates. Prior (grey) and posterior (black) distributions are shown for the density-independent mortality of the 0-group () and ages 1–3 () and posterior distributions (uniform priors) are shown for the density-dependent mortality rates of ages 1–3 (, , ). Figure S6: Parameter estimates for the age-specific fishing mortality. Figure S7: Estimated year-effect of the fishing mortality. Figure S8: Abundance trends for eggs, larvae, 0-group cod, and age-classes 1–9. Shown are the observations (black), corrected for age-specific catchability, and the model predictions (grey) with 95% credible intervals. Note the log-scale and the different periods for the abundance time-series corresponding to the actual observations (eggs/larvae: 1959–1990; 0-group: 1966–2010; ages 1–9: 1981–2010). Figure S9: Estimates of age-specific observation errors of the Barents Sea survey. Figure S10: Estimates of age-specific catchabilities of the Barents Sea survey. For age-classes 1 and 2 surveyability was independently estimated for the period before 1993 (open circles). (ZIP) [file pone.0098940.s001.zip › Ohlberger et al Supporting Information/Figure S1.pdf]

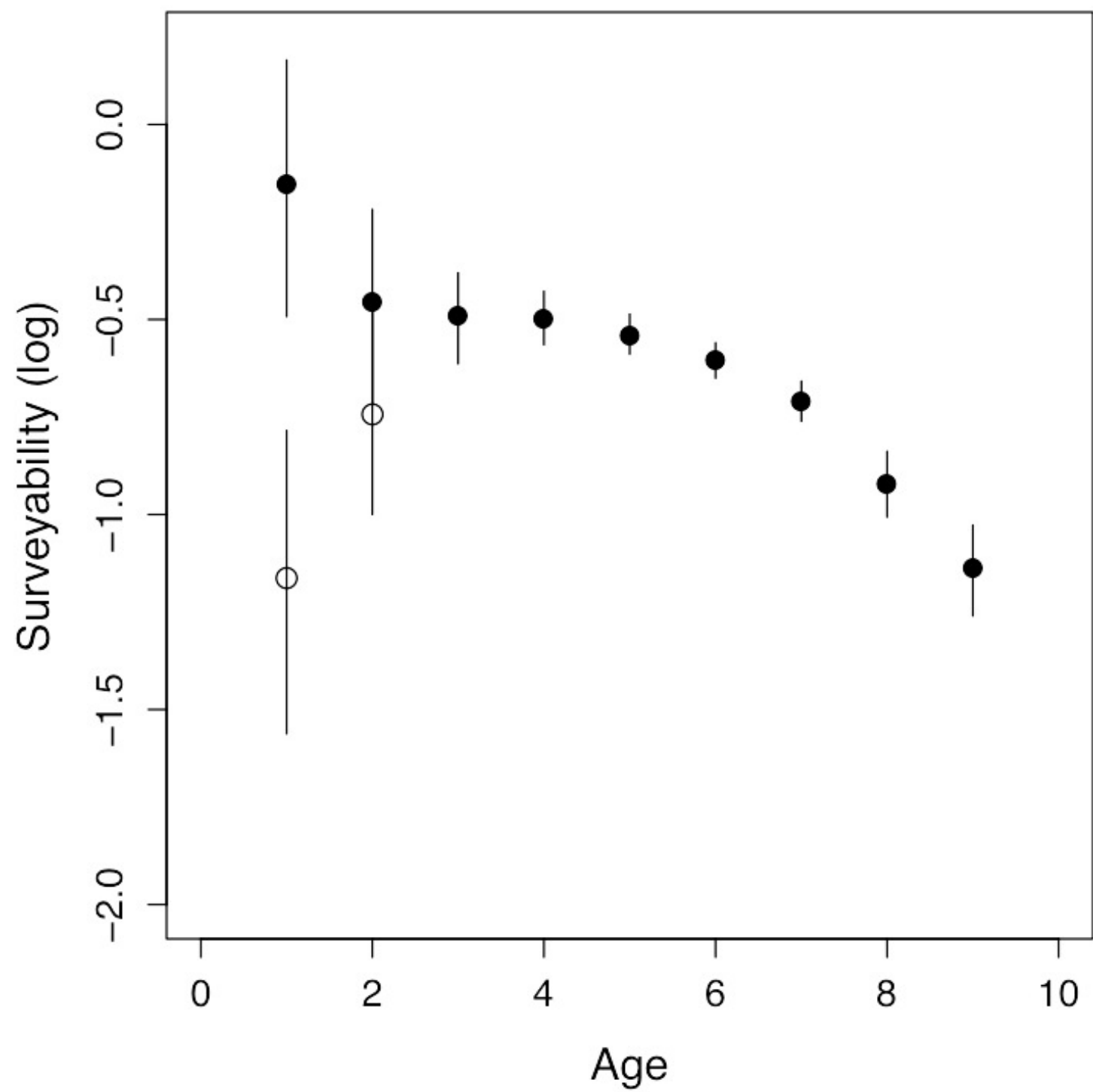

Supplement: File S1 — Contains the files: Text S1: Model tests and extensions. Table S1: Posterior medians and 95% credible intervals for all model parameters. Figure S1: Posterior distributions of all model parameters. Figure S2: Temporal autocorrelation of all model parameters. Figure S3: Cross-correlations of all model parameters. Figure S4: Estimated process errors. Shown are the process errors for spawning () and early juvenile mortality (). Figure S5: Prior and posterior distributions of mortality rates. Prior (grey) and posterior (black) distributions are shown for the density-independent mortality of the 0-group () and ages 1–3 () and posterior distributions (uniform priors) are shown for the density-dependent mortality rates of ages 1–3 (, , ). Figure S6: Parameter estimates for the age-specific fishing mortality. Figure S7: Estimated year-effect of the fishing mortality. Figure S8: Abundance trends for eggs, larvae, 0-group cod, and age-classes 1–9. Shown are the observations (black), corrected for age-specific catchability, and the model predictions (grey) with 95% credible intervals. Note the log-scale and the different periods for the abundance time-series corresponding to the actual observations (eggs/larvae: 1959–1990; 0-group: 1966–2010; ages 1–9: 1981–2010). Figure S9: Estimates of age-specific observation errors of the Barents Sea survey. Figure S10: Estimates of age-specific catchabilities of the Barents Sea survey. For age-classes 1 and 2 surveyability was independently estimated for the period before 1993 (open circles). (ZIP) [file pone.0098940.s001.zip › Ohlberger et al Supporting Information/Figure S10.pdf]

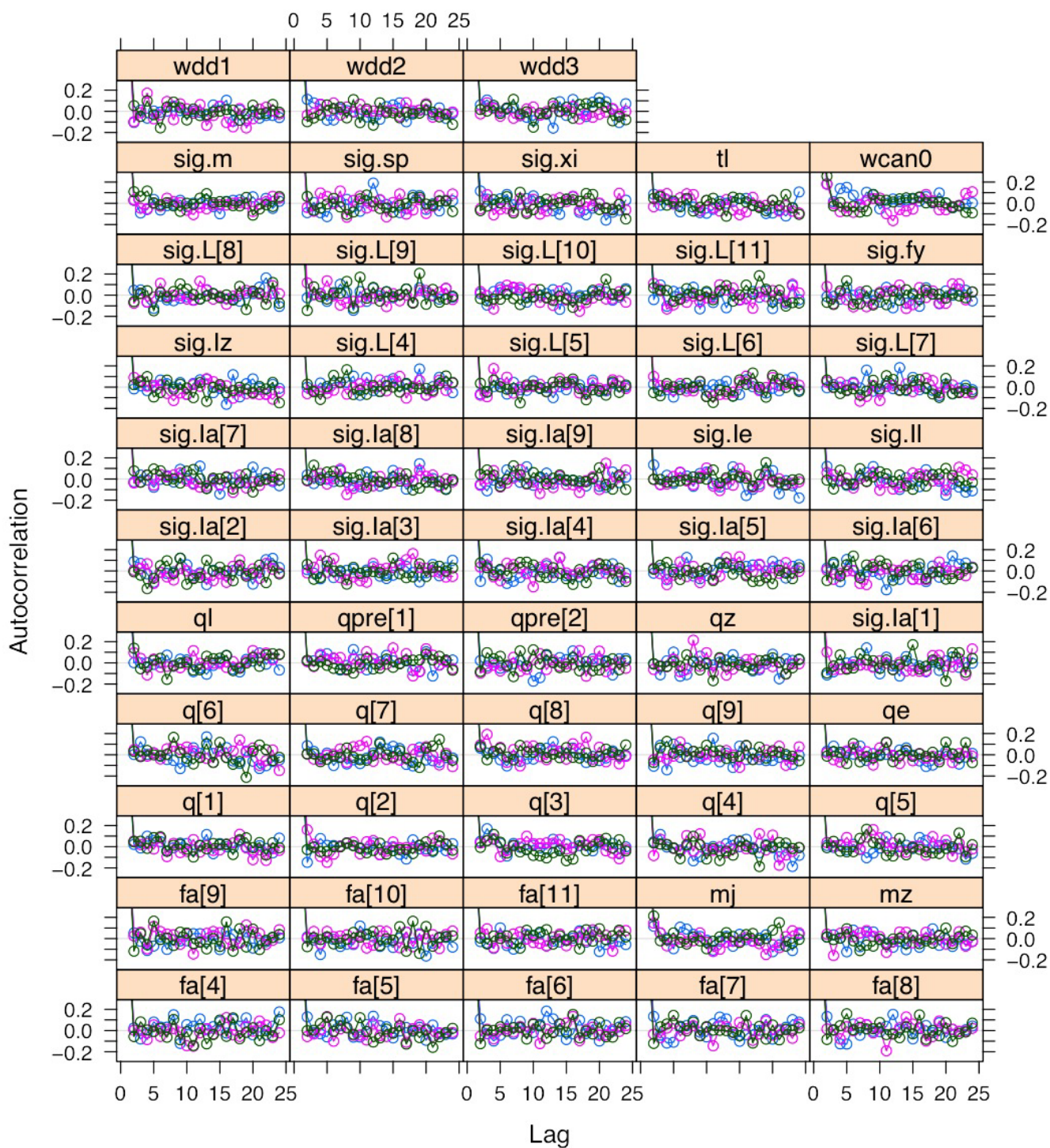

Supplement: File S1 — Contains the files: Text S1: Model tests and extensions. Table S1: Posterior medians and 95% credible intervals for all model parameters. Figure S1: Posterior distributions of all model parameters. Figure S2: Temporal autocorrelation of all model parameters. Figure S3: Cross-correlations of all model parameters. Figure S4: Estimated process errors. Shown are the process errors for spawning () and early juvenile mortality (). Figure S5: Prior and posterior distributions of mortality rates. Prior (grey) and posterior (black) distributions are shown for the density-independent mortality of the 0-group () and ages 1–3 () and posterior distributions (uniform priors) are shown for the density-dependent mortality rates of ages 1–3 (, , ). Figure S6: Parameter estimates for the age-specific fishing mortality. Figure S7: Estimated year-effect of the fishing mortality. Figure S8: Abundance trends for eggs, larvae, 0-group cod, and age-classes 1–9. Shown are the observations (black), corrected for age-specific catchability, and the model predictions (grey) with 95% credible intervals. Note the log-scale and the different periods for the abundance time-series corresponding to the actual observations (eggs/larvae: 1959–1990; 0-group: 1966–2010; ages 1–9: 1981–2010). Figure S9: Estimates of age-specific observation errors of the Barents Sea survey. Figure S10: Estimates of age-specific catchabilities of the Barents Sea survey. For age-classes 1 and 2 surveyability was independently estimated for the period before 1993 (open circles). (ZIP) [file pone.0098940.s001.zip › Ohlberger et al Supporting Information/Figure S2.pdf]

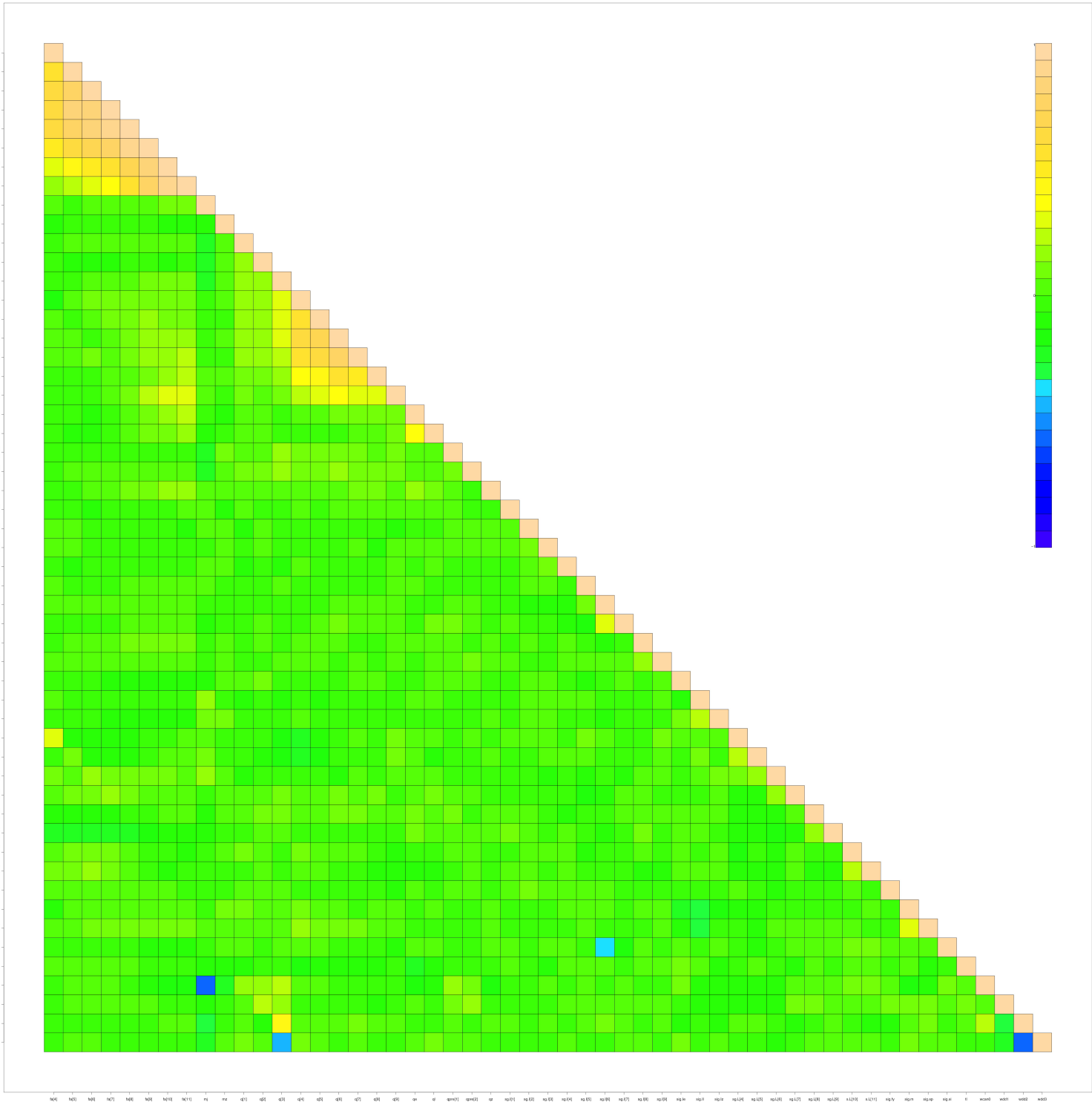

Supplement: File S1 — Contains the files: Text S1: Model tests and extensions. Table S1: Posterior medians and 95% credible intervals for all model parameters. Figure S1: Posterior distributions of all model parameters. Figure S2: Temporal autocorrelation of all model parameters. Figure S3: Cross-correlations of all model parameters. Figure S4: Estimated process errors. Shown are the process errors for spawning () and early juvenile mortality (). Figure S5: Prior and posterior distributions of mortality rates. Prior (grey) and posterior (black) distributions are shown for the density-independent mortality of the 0-group () and ages 1–3 () and posterior distributions (uniform priors) are shown for the density-dependent mortality rates of ages 1–3 (, , ). Figure S6: Parameter estimates for the age-specific fishing mortality. Figure S7: Estimated year-effect of the fishing mortality. Figure S8: Abundance trends for eggs, larvae, 0-group cod, and age-classes 1–9. Shown are the observations (black), corrected for age-specific catchability, and the model predictions (grey) with 95% credible intervals. Note the log-scale and the different periods for the abundance time-series corresponding to the actual observations (eggs/larvae: 1959–1990; 0-group: 1966–2010; ages 1–9: 1981–2010). Figure S9: Estimates of age-specific observation errors of the Barents Sea survey. Figure S10: Estimates of age-specific catchabilities of the Barents Sea survey. For age-classes 1 and 2 surveyability was independently estimated for the period before 1993 (open circles). (ZIP) [file pone.0098940.s001.zip › Ohlberger et al Supporting Information/Figure S3.pdf]

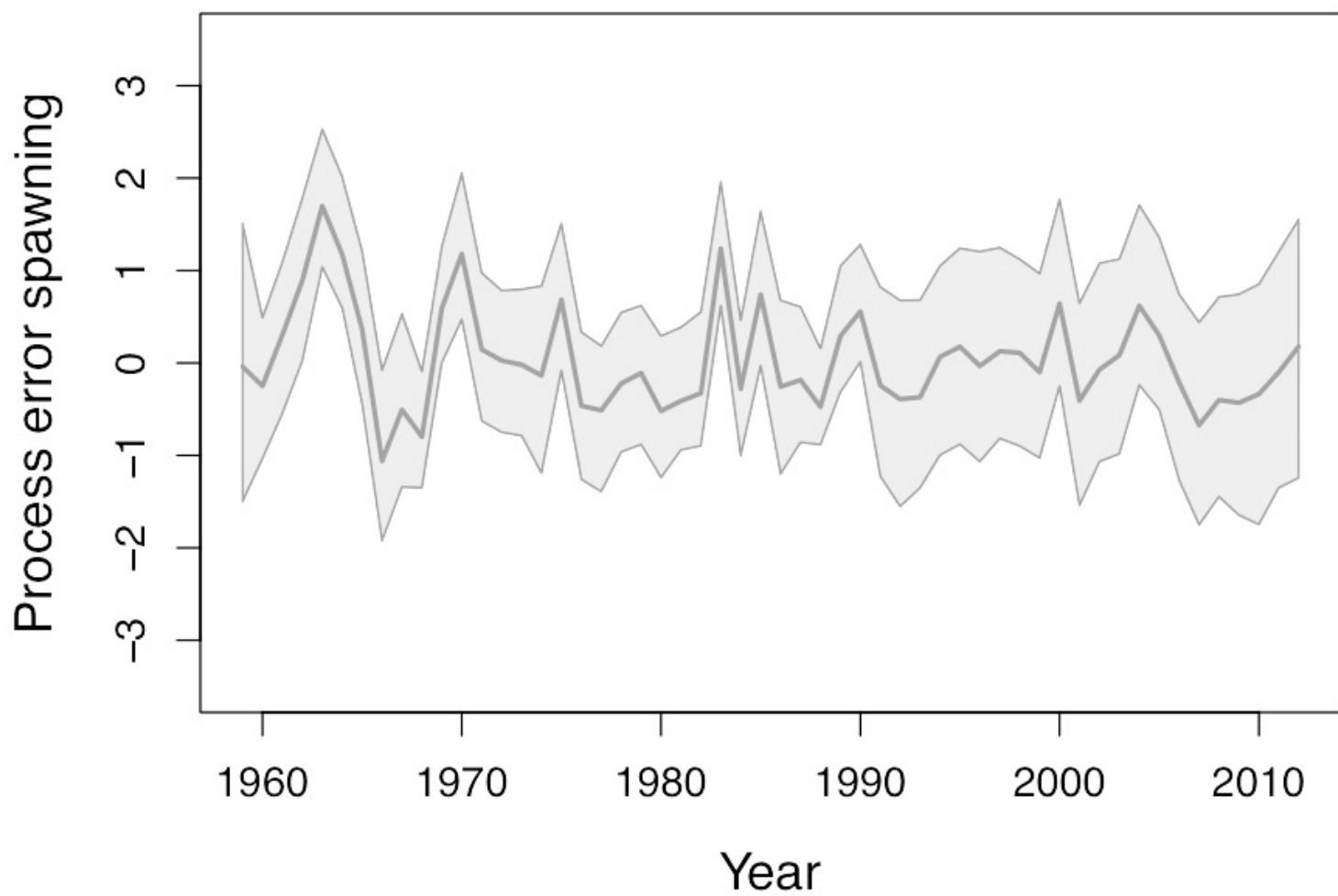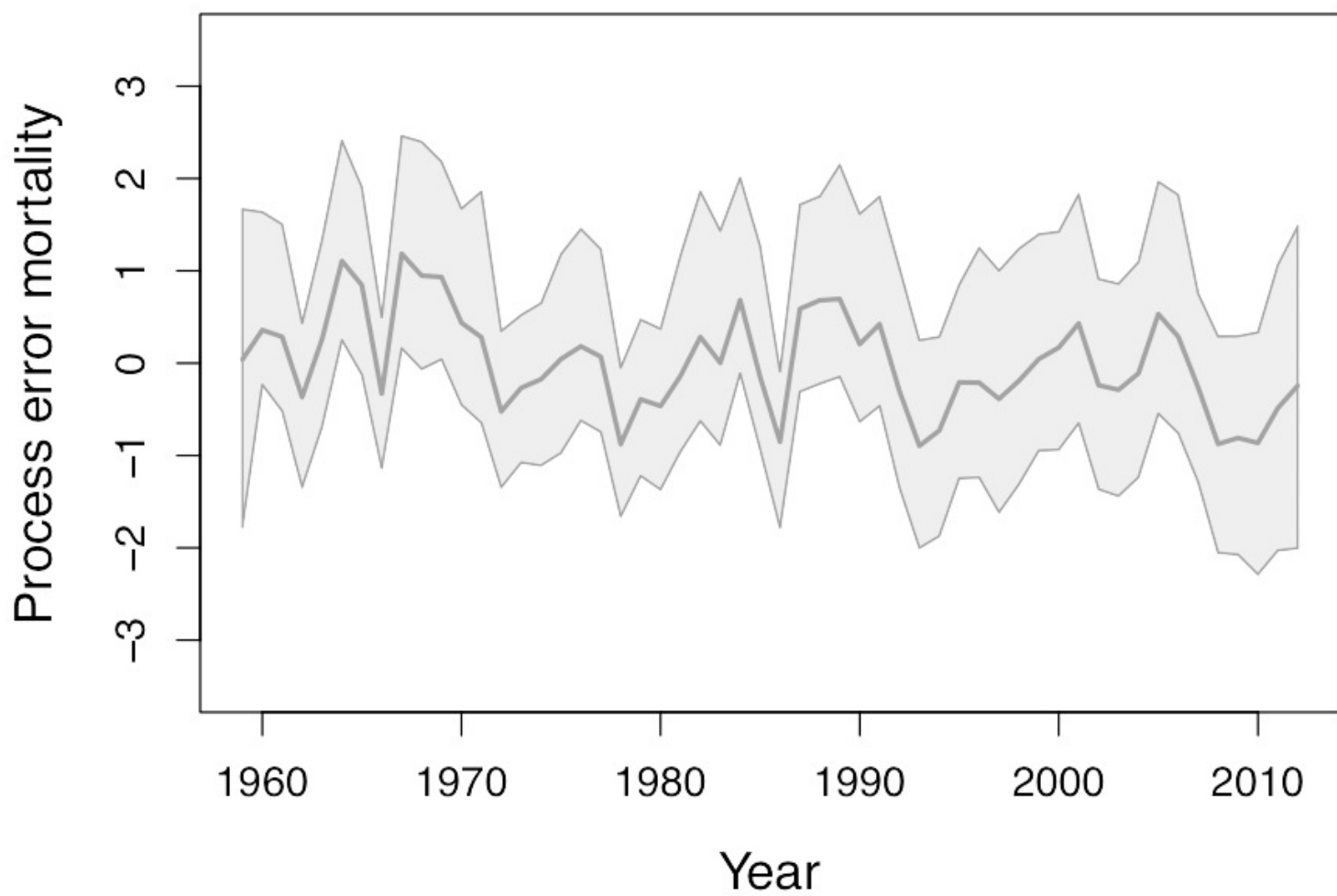

Supplement: File S1 — Contains the files: Text S1: Model tests and extensions. Table S1: Posterior medians and 95% credible intervals for all model parameters. Figure S1: Posterior distributions of all model parameters. Figure S2: Temporal autocorrelation of all model parameters. Figure S3: Cross-correlations of all model parameters. Figure S4: Estimated process errors. Shown are the process errors for spawning () and early juvenile mortality (). Figure S5: Prior and posterior distributions of mortality rates. Prior (grey) and posterior (black) distributions are shown for the density-independent mortality of the 0-group () and ages 1–3 () and posterior distributions (uniform priors) are shown for the density-dependent mortality rates of ages 1–3 (, , ). Figure S6: Parameter estimates for the age-specific fishing mortality. Figure S7: Estimated year-effect of the fishing mortality. Figure S8: Abundance trends for eggs, larvae, 0-group cod, and age-classes 1–9. Shown are the observations (black), corrected for age-specific catchability, and the model predictions (grey) with 95% credible intervals. Note the log-scale and the different periods for the abundance time-series corresponding to the actual observations (eggs/larvae: 1959–1990; 0-group: 1966–2010; ages 1–9: 1981–2010). Figure S9: Estimates of age-specific observation errors of the Barents Sea survey. Figure S10: Estimates of age-specific catchabilities of the Barents Sea survey. For age-classes 1 and 2 surveyability was independently estimated for the period before 1993 (open circles). (ZIP) [file pone.0098940.s001.zip › Ohlberger et al Supporting Information/Figure S4.pdf]

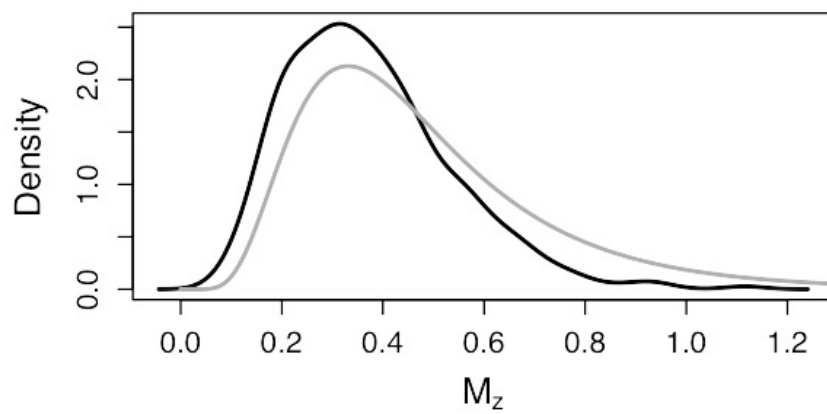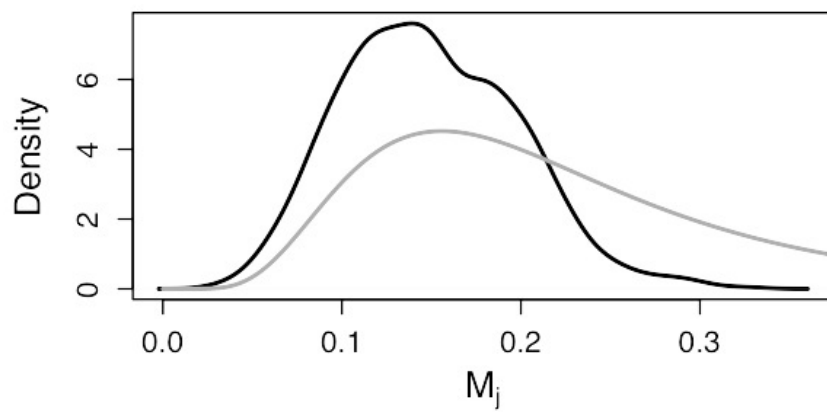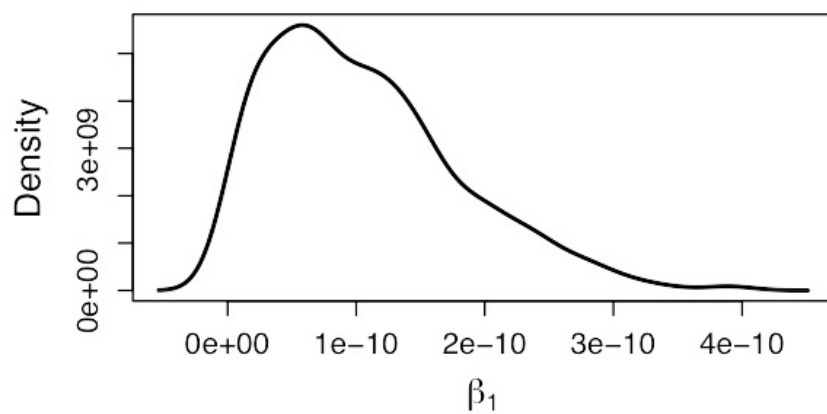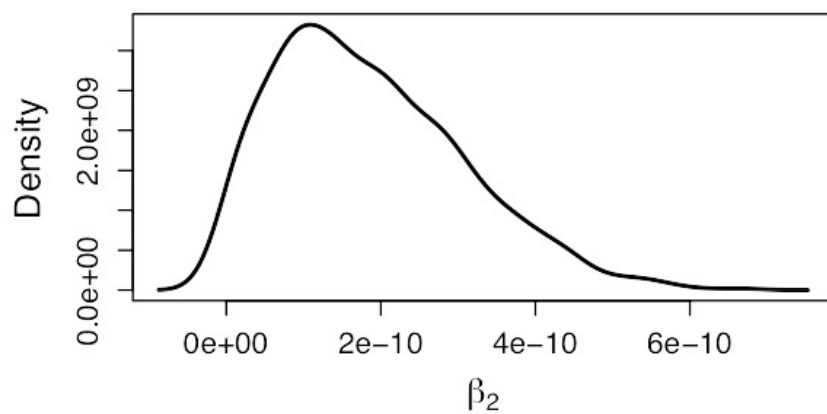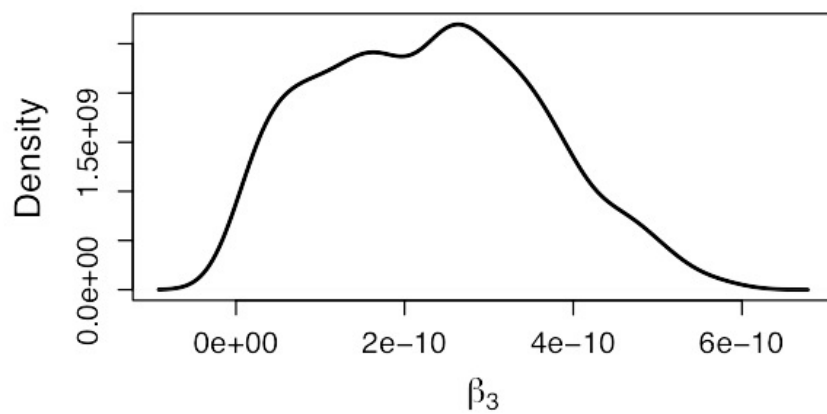

Supplement: File S1 — Contains the files: Text S1: Model tests and extensions. Table S1: Posterior medians and 95% credible intervals for all model parameters. Figure S1: Posterior distributions of all model parameters. Figure S2: Temporal autocorrelation of all model parameters. Figure S3: Cross-correlations of all model parameters. Figure S4: Estimated process errors. Shown are the process errors for spawning () and early juvenile mortality (). Figure S5: Prior and posterior distributions of mortality rates. Prior (grey) and posterior (black) distributions are shown for the density-independent mortality of the 0-group () and ages 1–3 () and posterior distributions (uniform priors) are shown for the density-dependent mortality rates of ages 1–3 (, , ). Figure S6: Parameter estimates for the age-specific fishing mortality. Figure S7: Estimated year-effect of the fishing mortality. Figure S8: Abundance trends for eggs, larvae, 0-group cod, and age-classes 1–9. Shown are the observations (black), corrected for age-specific catchability, and the model predictions (grey) with 95% credible intervals. Note the log-scale and the different periods for the abundance time-series corresponding to the actual observations (eggs/larvae: 1959–1990; 0-group: 1966–2010; ages 1–9: 1981–2010). Figure S9: Estimates of age-specific observation errors of the Barents Sea survey. Figure S10: Estimates of age-specific catchabilities of the Barents Sea survey. For age-classes 1 and 2 surveyability was independently estimated for the period before 1993 (open circles). (ZIP) [file pone.0098940.s001.zip › Ohlberger et al Supporting Information/Figure S5.pdf]

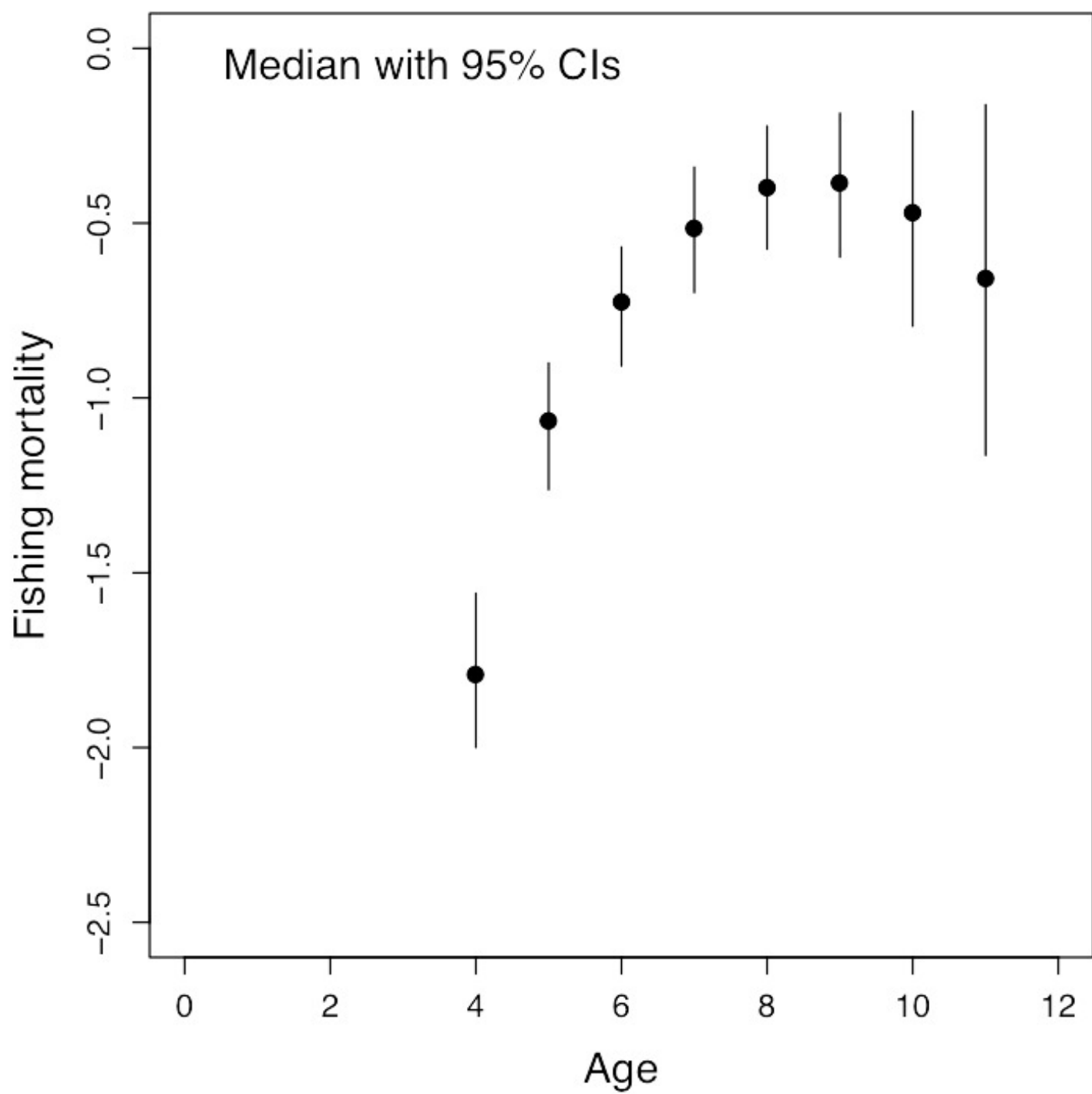

Supplement: File S1 — Contains the files: Text S1: Model tests and extensions. Table S1: Posterior medians and 95% credible intervals for all model parameters. Figure S1: Posterior distributions of all model parameters. Figure S2: Temporal autocorrelation of all model parameters. Figure S3: Cross-correlations of all model parameters. Figure S4: Estimated process errors. Shown are the process errors for spawning () and early juvenile mortality (). Figure S5: Prior and posterior distributions of mortality rates. Prior (grey) and posterior (black) distributions are shown for the density-independent mortality of the 0-group () and ages 1–3 () and posterior distributions (uniform priors) are shown for the density-dependent mortality rates of ages 1–3 (, , ). Figure S6: Parameter estimates for the age-specific fishing mortality. Figure S7: Estimated year-effect of the fishing mortality. Figure S8: Abundance trends for eggs, larvae, 0-group cod, and age-classes 1–9. Shown are the observations (black), corrected for age-specific catchability, and the model predictions (grey) with 95% credible intervals. Note the log-scale and the different periods for the abundance time-series corresponding to the actual observations (eggs/larvae: 1959–1990; 0-group: 1966–2010; ages 1–9: 1981–2010). Figure S9: Estimates of age-specific observation errors of the Barents Sea survey. Figure S10: Estimates of age-specific catchabilities of the Barents Sea survey. For age-classes 1 and 2 surveyability was independently estimated for the period before 1993 (open circles). (ZIP) [file pone.0098940.s001.zip › Ohlberger et al Supporting Information/Figure S6.pdf]

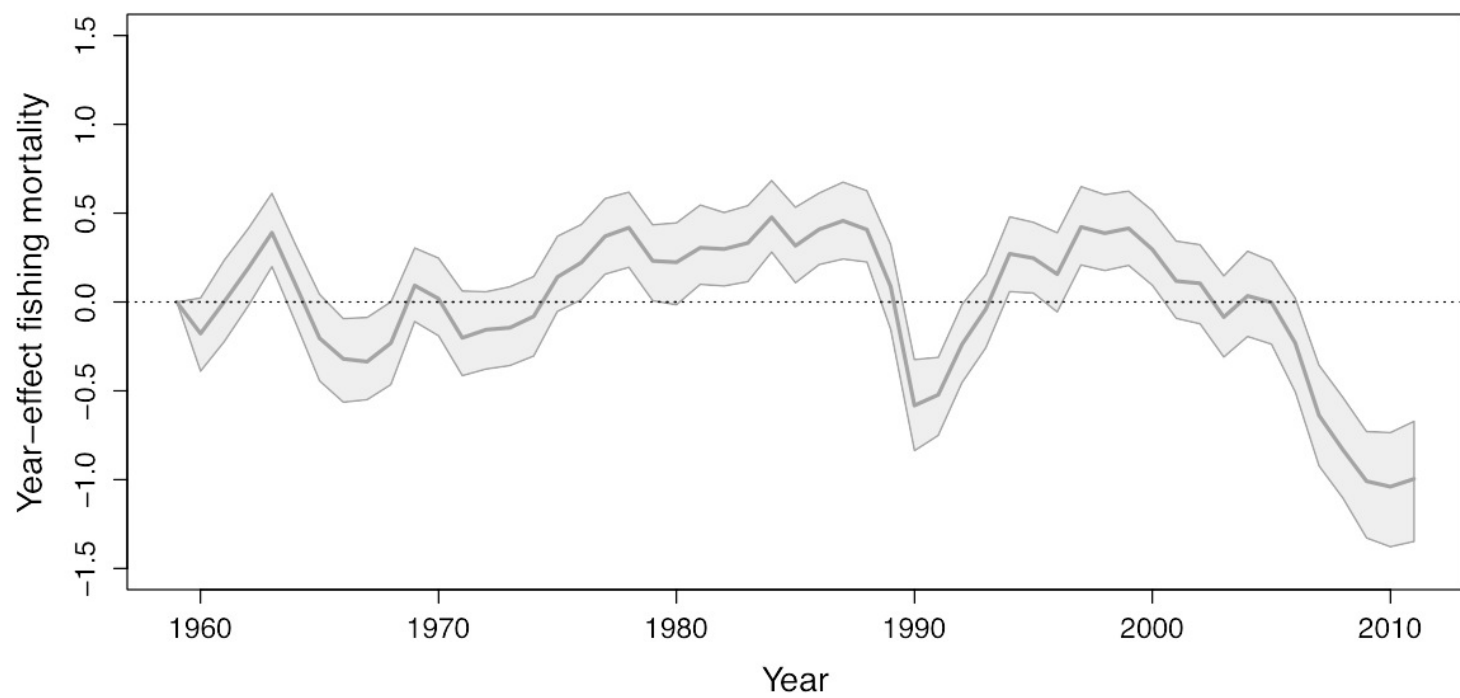

Supplement: File S1 — Contains the files: Text S1: Model tests and extensions. Table S1: Posterior medians and 95% credible intervals for all model parameters. Figure S1: Posterior distributions of all model parameters. Figure S2: Temporal autocorrelation of all model parameters. Figure S3: Cross-correlations of all model parameters. Figure S4: Estimated process errors. Shown are the process errors for spawning () and early juvenile mortality (). Figure S5: Prior and posterior distributions of mortality rates. Prior (grey) and posterior (black) distributions are shown for the density-independent mortality of the 0-group () and ages 1–3 () and posterior distributions (uniform priors) are shown for the density-dependent mortality rates of ages 1–3 (, , ). Figure S6: Parameter estimates for the age-specific fishing mortality. Figure S7: Estimated year-effect of the fishing mortality. Figure S8: Abundance trends for eggs, larvae, 0-group cod, and age-classes 1–9. Shown are the observations (black), corrected for age-specific catchability, and the model predictions (grey) with 95% credible intervals. Note the log-scale and the different periods for the abundance time-series corresponding to the actual observations (eggs/larvae: 1959–1990; 0-group: 1966–2010; ages 1–9: 1981–2010). Figure S9: Estimates of age-specific observation errors of the Barents Sea survey. Figure S10: Estimates of age-specific catchabilities of the Barents Sea survey. For age-classes 1 and 2 surveyability was independently estimated for the period before 1993 (open circles). (ZIP) [file pone.0098940.s001.zip › Ohlberger et al Supporting Information/Figure S7.pdf]

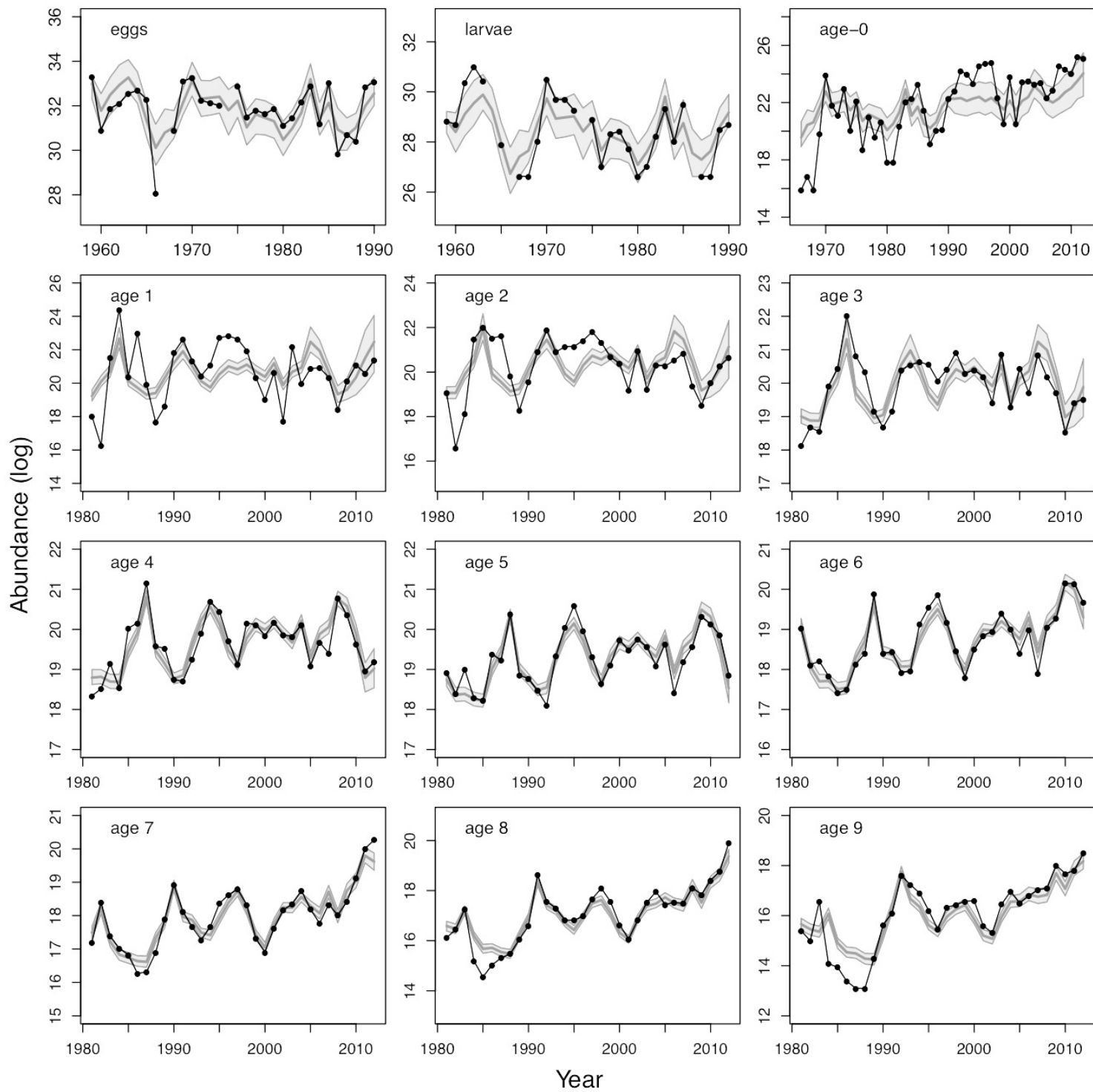

Supplement: File S1 — Contains the files: Text S1: Model tests and extensions. Table S1: Posterior medians and 95% credible intervals for all model parameters. Figure S1: Posterior distributions of all model parameters. Figure S2: Temporal autocorrelation of all model parameters. Figure S3: Cross-correlations of all model parameters. Figure S4: Estimated process errors. Shown are the process errors for spawning () and early juvenile mortality (). Figure S5: Prior and posterior distributions of mortality rates. Prior (grey) and posterior (black) distributions are shown for the density-independent mortality of the 0-group () and ages 1–3 () and posterior distributions (uniform priors) are shown for the density-dependent mortality rates of ages 1–3 (, , ). Figure S6: Parameter estimates for the age-specific fishing mortality. Figure S7: Estimated year-effect of the fishing mortality. Figure S8: Abundance trends for eggs, larvae, 0-group cod, and age-classes 1–9. Shown are the observations (black), corrected for age-specific catchability, and the model predictions (grey) with 95% credible intervals. Note the log-scale and the different periods for the abundance time-series corresponding to the actual observations (eggs/larvae: 1959–1990; 0-group: 1966–2010; ages 1–9: 1981–2010). Figure S9: Estimates of age-specific observation errors of the Barents Sea survey. Figure S10: Estimates of age-specific catchabilities of the Barents Sea survey. For age-classes 1 and 2 surveyability was independently estimated for the period before 1993 (open circles). (ZIP) [file pone.0098940.s001.zip › Ohlberger et al Supporting Information/Figure S8.pdf]

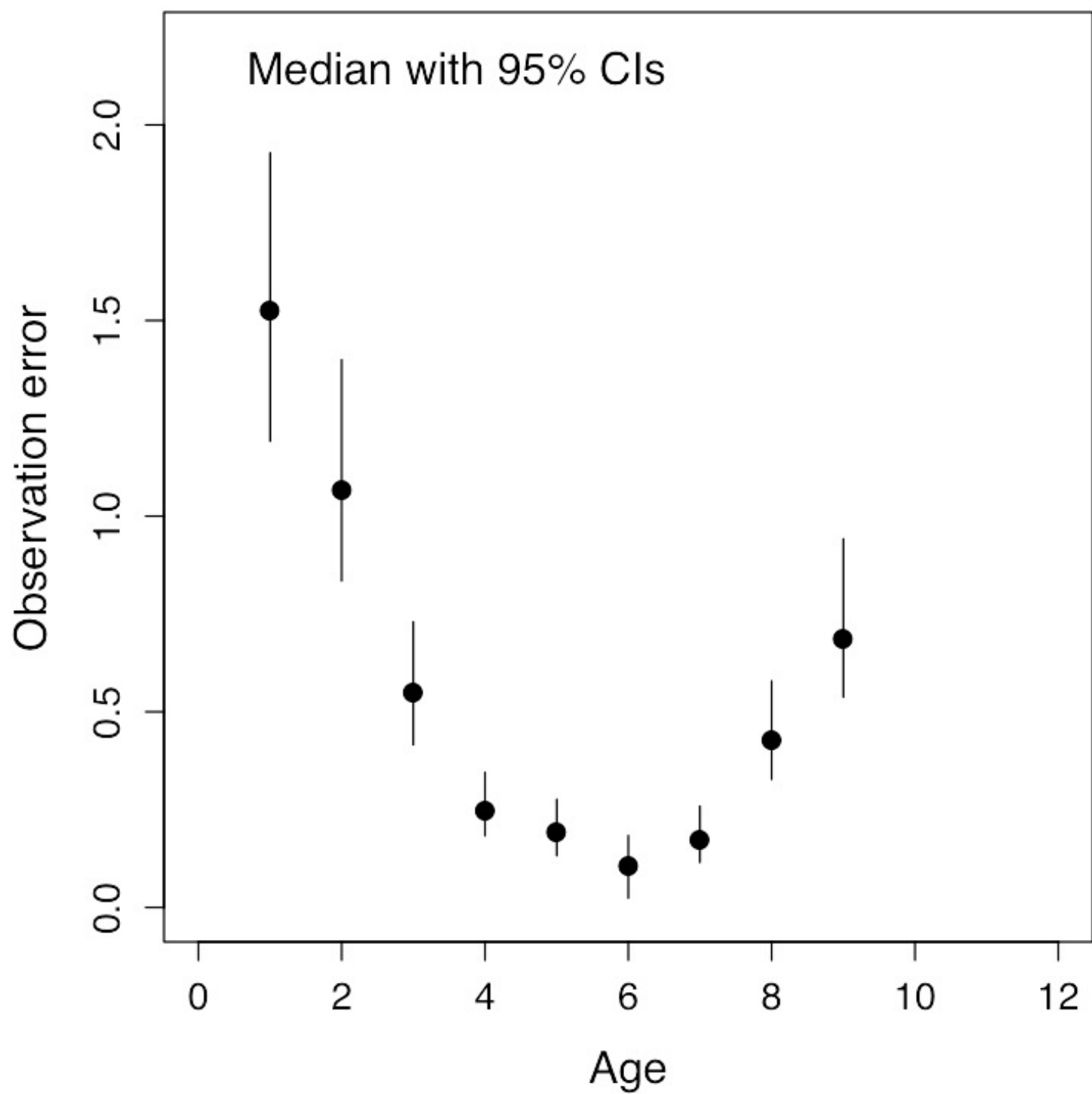

Supplement: File S1 — Contains the files: Text S1: Model tests and extensions. Table S1: Posterior medians and 95% credible intervals for all model parameters. Figure S1: Posterior distributions of all model parameters. Figure S2: Temporal autocorrelation of all model parameters. Figure S3: Cross-correlations of all model parameters. Figure S4: Estimated process errors. Shown are the process errors for spawning () and early juvenile mortality (). Figure S5: Prior and posterior distributions of mortality rates. Prior (grey) and posterior (black) distributions are shown for the density-independent mortality of the 0-group () and ages 1–3 () and posterior distributions (uniform priors) are shown for the density-dependent mortality rates of ages 1–3 (, , ). Figure S6: Parameter estimates for the age-specific fishing mortality. Figure S7: Estimated year-effect of the fishing mortality. Figure S8: Abundance trends for eggs, larvae, 0-group cod, and age-classes 1–9. Shown are the observations (black), corrected for age-specific catchability, and the model predictions (grey) with 95% credible intervals. Note the log-scale and the different periods for the abundance time-series corresponding to the actual observations (eggs/larvae: 1959–1990; 0-group: 1966–2010; ages 1–9: 1981–2010). Figure S9: Estimates of age-specific observation errors of the Barents Sea survey. Figure S10: Estimates of age-specific catchabilities of the Barents Sea survey. For age-classes 1 and 2 surveyability was independently estimated for the period before 1993 (open circles). (ZIP) [file pone.0098940.s001.zip › Ohlberger et al Supporting Information/Figure S9.pdf]
